# Divergence-Conforming Velocity and Vorticity Approximations for Incompressible Fluids Obtained with Minimal Facet Coupling

**DOI:** 10.1007/s10915-023-02203-8

**Published:** 2023-05-11

**Authors:** J. Gopalakrishnan, L. Kogler, P. L. Lederer, J. Schöberl

**Affiliations:** 1 Portland State University, PO Box 751, Portland, OR 97207 USA; 2 Institute for Analysis and Scientific Computing, TU Wien, Wiedner Hauptstraße 8-10, 1040 Wien, Austria; 3 Department of Applied Mathematics, University of Twente, Hallenweg 19, 7522 NH Enschede, Netherlands

**Keywords:** Incompressible Stokes equations, Mixed finite elements, Pressure-robustness, Hybrid discontinuous Galerkin methods, Discrete Korn inequality

## Abstract

We introduce two new lowest order methods, a mixed method, and a hybrid discontinuous Galerkin method, for the approximation of incompressible flows. Both methods use divergence-conforming linear Brezzi–Douglas–Marini space for approximating the velocity and the lowest order Raviart–Thomas space for approximating the vorticity. Our methods are based on the physically correct viscous stress tensor of the fluid, involving the symmetric gradient of velocity (rather than the gradient), provide exactly divergence-free discrete velocity solutions, and optimal error estimates that are also pressure robust. We explain how the methods are constructed using the minimal number of coupling degrees of freedom per facet. The stability analysis of both methods are based on a Korn-like inequality for vector finite elements with continuous normal component. Numerical examples illustrate the theoretical findings and offer comparisons of condition numbers between the two new methods.

## Introduction

In this work we introduce two new methods for the discretization of the steady incompressible Stokes equations in three space dimensions. Let $\varOmega \subset {\mathbb {R}}^3$ be an open bounded domain with Lipschitz boundary $\partial \varOmega$ that is split into the Dirichlet boundary $\varGamma _D$ and outflow boundary $\varGamma _N$. The Stokes system for the fluid *velocity* *u* and the *pressure* *p* is given by

1a $$\begin{aligned} -{\text {div}}(\nu \varepsilon (u)) + \nabla p&= f{} & {} \quad \text {in } \varOmega , \end{aligned}$$

1b $$\begin{aligned} {\text {div}}u&=0{} & {} \quad \text {in } \varOmega , \end{aligned}$$

1c $$\begin{aligned} u&= 0{} & {} \quad \text {on } \varGamma _D, \end{aligned}$$

1d $$\begin{aligned} (-\nu \varepsilon (u) + p I)n&= 0{} & {} \quad \text {on } \varGamma _N, \end{aligned}$$

where $\varepsilon (u):= (\nabla u + \nabla u ^{{\text {T}}})/2$ is the symmetric gradient, $f: \varOmega \rightarrow {\mathbb {R}}^3$ is an external body force, $\nu$ is twice the kinematic viscosity, *n* is the outward unit normal vector and $I\in {\mathbb {R}}^{3\times 3}$ is the identity matrix. We assume that both $\varGamma _D$ and $\varGamma _N$ have positive boundary measure, and any rigid displacement vanishing on $\varGamma _D$ vanishes everywhere in $\varOmega$. (As usual, when $\varGamma _N$ is empty the pressure space must be adapted to obtain a unique pressure [18], but we omit this case for simplicity.) Next, define the *viscous stress tensor* [23] by $\sigma = \nu \varepsilon (u)$ and the *vorticity* by $\omega = {\text {curl}}u$. Using them, we can rewrite the above system as

2a $$\begin{aligned} \nu ^{-1}{{\text {dev}}{\sigma }} - \nabla u + \kappa (\omega )&= 0{} & {} \quad \text {in } \varOmega , \end{aligned}$$

2b $$\begin{aligned} -{\text {div}}\sigma + \nabla p&= f{} & {} \quad \text {in } \varOmega , \end{aligned}$$

2c $$\begin{aligned} \sigma - \sigma ^{{\text {T}}}&= 0{} & {} \quad \text {in } \varOmega , \end{aligned}$$

2d $$\begin{aligned} {\text {div}}u&=0{} & {} \quad \text {in } \varOmega , \end{aligned}$$

2e $$\begin{aligned} u&= 0{} & {} \quad \text {on } \varGamma _D, \end{aligned}$$

2f $$\begin{aligned} (\sigma - p I)n&= 0{} & {} \quad \text {on } \varGamma _N. \end{aligned}$$

Here we used the deviatoric part of the tensor $\tau$ given by ${\text {dev}}{\tau }:= \tau - \frac{1}{3}{{\text {tr}}(\tau )} I,$ the matrix trace ${{\text {tr}}(\tau )}:= \sum _{i=1}^3 \tau _{ii}$, and the operator $\kappa : {\mathbb {R}}^{3} \rightarrow \{\tau \in {\mathbb {R}}^{3 \times 3}: \tau + \tau ^{{{\text {T}}}} = 0 \}$ defined by

$$\begin{aligned} \kappa (v) = \frac{1}{2} \begin{pmatrix} 0 &{} \quad -v_3 &{} \quad v_2 \\ v_3 &{} \quad 0 &{} \quad -v_1 \\ -v_2 &{} \quad v_1 &{} \quad 0 \end{pmatrix}. \end{aligned}$$

Note the obvious identities

3 $$\begin{aligned} \nabla v = \varepsilon (v) + \kappa ({\text {curl}}v), \qquad 2 \kappa (v) w = v \times w, \end{aligned}$$

for vector fields *v* and *w* (the first of which was already used in (2a)). We will refer to system (1) as the primal formulation and system (2) as the mixed formulation.

The literature on discretizations of (1) and (2) is too vast to list here. The relatively recent quest for exactly divergence-free velocity solutions and pressure-independent a priori error estimates for velocity, often referred to as pressure robust estimates [27, 30], has rejuvenated the field. A recurring theme in this vast literature, from the early non-conforming method of [10] to the more recent [29], is the desire to improve computational efficiency by minimizing inter-element coupling. However, less studied are its side effects on stability when the actual physical flux replaces the often-used simplified diffusive flux, i.e., when

4 $$\begin{aligned} -{\text {div}}(\nu \varepsilon (u)) \quad \text {replaces} \quad -{\text {div}}(\nu \nabla u), \end{aligned}$$

even though an early work [11] cautions how the lowest order method of [10] can become unstable when doing so. Such instabilities arise because the larger null space of $\varepsilon$ necessitates increased inter-element coupling (as explained in more detail below) and are manifested in certain lowest order cases with insufficient inter-element coupling. In this work, focusing on the lowest order case, we identify new stable finite element methods, with the minimal necessary inter-element coupling, that yield exactly divergence-free and pressure robust velocities. New methods based on both the primal and the mixed formulations are designed.

Yet another reason for focusing on the lowest order case is its utility in preconditioning. Roughly speaking, a common strategy for preconditioning high order Stokes discretizations involves combining local (high order) error dampers via, say block Jacobi or other smoothers, with a global (low order) error corrector such as multigrid (or even a direct solver) applied to the smaller lowest order discretization. From this point of view, it is desirable to have stable low order versions (that remain stable under (4)) of high order methods for design of preconditioners, an interesting topic which we shall not touch upon further in this paper.

To delve deeper into the mechanics of the above-mentioned instability, consider the kernel of $\varepsilon$, consisting of rigid displacements of the form $x\rightarrow a + b \times x$ with $a,b\in {\mathbb {R}}^3$. Reasonable methods approximating the operator $-{\text {div}}(\nu \varepsilon (u))$ produce element matrices whose nullspaces contain these rigid displacements. Ideally, when these element-wise rigid displacements are subjected to the inter-element continuity conditions of the discrete velocity space, they should equal element-wise restrictions of a global rigid displacement on $\varOmega$ (which can then be eliminated by boundary conditions). However, if the inter-element coupling in the discrete velocity space is so weak to allow for the existence of a *u* in it that does not equal a global rigid displacement on $\varOmega$ even though $u|_T$ is a rigid displacement on every mesh element *T*, then instabilities can arise [11].

The discrete velocity space we have in mind is the lowest order (piecewise linear) $H({\text {div}})$-conforming Brezzi-Douglas-Marini ($\mathcal {BDM}^1$) space [3]. (A basic premise of this paper is the unquestionable utility of $H({\text {div}})$-conforming velocity spaces to obtain exactly divergence-free discrete Stokes velocity fields, well established in prior works [8, 9, 20, 21, 29]). Hence, to understand how to avoid the above-mentioned instability while setting velocity in the $\mathcal {BDM}^1$ space, we ask the following question: *how many coupling degrees of freedom* (dofs) *are needed to guarantee that two rigid displacements* $u_\pm$,*given respectively on two adjacent elements *$T_\pm$, *coincide on the common interface *$F = \partial T_+ \cap \partial T_-$?

**Fig. 1:**
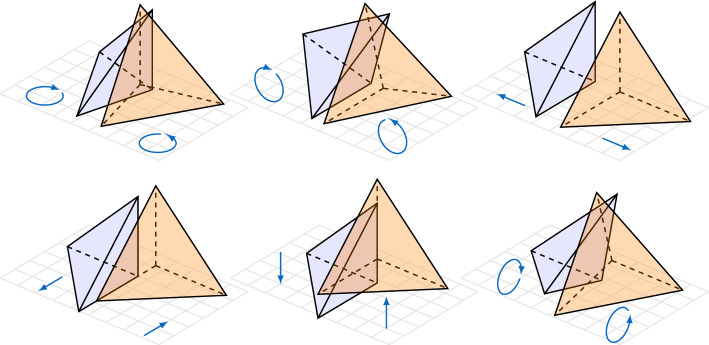
Configurations of adjacent elements after deformation by piecewise rigid displacements of two adjacent elements $T_\pm$

**Fig. 2:**
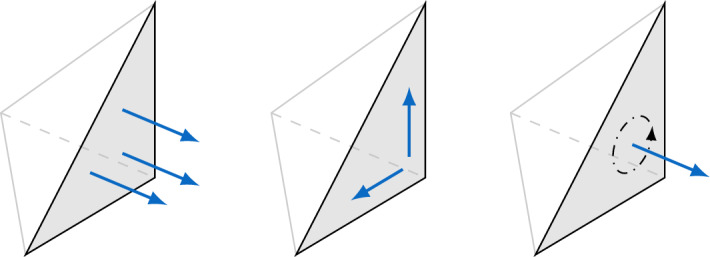
Classification of facet dofs in our new methods into three types: (1) normal velocity components in the form of $\mathcal {BDM}^1$ facet dofs, (2) tangential facet velocities, (3) normal vorticity as ${\mathcal {R}}{\mathcal {T}}^0$ facet dof

The pictorial representations of the deformations created by $u_\pm$ in Fig. 1 lead to the answer. Three of the pictured deformations are just translations (generated by the *a*-vector in $a + b \times x$). For a unit vector *b*, letting $R^b_\theta$ denote the unitary operator that performs a counterclockwise rotation by angle $\theta$ around *b*, it is easy to see that $R^b_\theta x = x + \theta (b \times x) + \mathcal {O}(\theta ^2)$ as $\theta \rightarrow 0$. Therefore the deformation created by the rigid displacement $b \times x$ can be viewed as an infinitesimal rotation about *b*. These deformations are portrayed in Fig. 1 as rotations about three linearly independent *b*-vectors. The first row in Fig. 1 illustrates deformations generated by piecewise rigid displacements which are given by two *b*-vectors coplanar with *F* and an *a*-vector normal to *F*. These rigid displacements are forbidden in the $\mathcal {BDM}^1$ space. Indeed, recall [3] that the $\mathcal {BDM}^1$ dofs on the facet *F* are given by the linear functionals $u \mapsto \int _F u \cdot n ~q \mathop {{\textrm{d}} s}$ for all linear polynomials *q* on *F*, where *n* is a normal vector on *F*. These represent three dofs illustrated in left diagram of Fig. 2. If these three dofs coincide for two rigid displacements $u_{\pm }$, then the corresponding normal component must be continuous on *F*. This continuity forbids the above-mentioned deformations to be generated by elements of the $\mathcal {BDM}^1$ space. We summarize this by saying that the rigid displacements portrayed in the first row of Fig. 1 are “controlled” by the three $\mathcal {BDM}^1$ dofs of the facet *F* which are illustrated in the left diagram of Fig. 2.

It remains to control the rigid displacements of the second row of Fig. 1 using three additional dofs per facet. To this end, our new methods have two additional spaces: (i) one that approximates the in-plane components of the velocity on facets, illustrated in the middle diagram of Fig. 2, used to control the first two rigid displacements in the second row of Fig. 1; and (ii) a second space, schematically indicated in the last diagram of Fig. 2, that controls the third deformation in the second row of Fig. 1. The latter deformation arises from piecewise rigid displacements of the form $u_\pm = b_\pm \times x$ with $b_\pm$ collinear to *n*, a unit normal of *F*. Since ${\text {curl}}(b_\pm \times x) = 2 b_\pm$, we can make the two rigid displacements coincide on *F* by requiring continuity of $n ldot {\text {curl}}u_\pm$. While continuity of $n \cdot {\text {curl}}u$ certainly holds if *u* is the exact Stokes velocity, it does not generally hold for *u* in $\mathcal {BDM}^1$. Hence, keeping in view that $\omega = {\text {curl}}u$ represents vorticity, *we incorporate this constraint in our new methods by approximating vorticity *$\omega$*in the lowest order Raviart-Thomas space.* This is our second additional space. Its single dof per facet is shown schematically in the last diagram of Fig. 2.

In the first part of the paper we will employ these additional spaces to construct a novel HDG method to approximate (1) and present a detailed stability and error analysis. HDG methods have become popular ever since its introduction in [7] which showed how interface variables, or facet variables, can be effectively used to construct DG schemes amenable to static condensation. In the method presented here, the interface variable approximates the tangential components of the velocity. The key technical ingredient in the analysis that reflects the insight garnered from the above pictorial discussion is a discrete Korn-like inequality for the $\mathcal {BDM}^1$ space (see Lemma 1 below, and the version with interface variables in Lemma 2).

The second part of this work discusses the derivation of a novel mixed method for the approximation of (2) and is motivated by our previous two papers [20, 21] and the many other works on discretizing (2) such as [12–15]. In [20] we derived the “Mass-Conserving Stress-yielding” (MCS) formulation where the symmetry of $\sigma$ was incorporated in a weak sense by means of a Lagrange multiplier that approximates $\omega = {\text {curl}}u$. While the $\omega$ there was approximated using element-wise linear (or higher degree) functions *without* any inter-element continuity requirements, the new mixed method we propose here will approximate $\omega$ in the lowest order Raviart-Thomas space instead. The lowest order case that was proved to be stable in [20] had nine coupling dofs per facet. We are able to reduce this number to the minimal six (the dimension of rigid displacements) in this paper. This minimal coupling was achieved earlier in [35] using a bubble-augmented velocity space which is a subspace of a degree-four vector polynomial space. Since higher degrees necessitate more expensive integration rules, we offer our simpler elements as an alternative.

Other methods that approximate the operator ${\text {div}}(\nu \nabla u)$, such as [10, 21, 29], are able to reduce the number of coupling dofs per facet even further. Since our focus here is on methods that approximate ${\text {div}}(\nu \varepsilon ( u))$, we restrict ourselves to a brief remark on this. Since the kernel of $\nabla$ (applied to vector fields) is three dimensional, we expect the minimal number of coupling dofs per facet to be three when approximating ${\text {div}}(\nu \nabla u)$. A method with this minimal coupling was achieved early by [10]. To also obtain pressure robust and exactly divergence-free solutions, prior works [21, 29] settled for a slightly higher five coupling dofs per facet in the lowest order case. It is now known that this can be improved by employing the technique of “relaxed $H({\text {div}}, \varOmega )$-conformity,” see [25, 26], which results in a method with the minimal three coupling dofs per facet and yet, thanks to a simple post-processing, provides optimal convergence orders and pressure robustness. While on the subject of coupling dofs, an explanation of our focus on three-dimensional (3D) domains is in order. On two-dimensional (2D) domains, the space of rigid displacements only has three dimensions. In the lowest order 2D case, $\mathcal {BDM}^1$ space provides two coupling dofs per facet edge, and the space of tangential facet velocities adds one more coupling degree of freedom. Thus the minimal facet coupling (of three dofs) needed to eliminate the rigid displacements are more immediate in 2D case when compared to the 3D case, which is why restrict to the 3D case henceforth.

The new HDG method and the new mixed method proposed in this paper both have the same coupling dofs, the same velocity convergence orders and the same structure preservation properties like pressure robustness and mass conservation. On closer comparison, two advantages of the mixed method are notable. One is its direct approximation of viscous stresses. Another is the absence of any stabilization parameters in it. In fact, in our numerical studies, the conditioning of a matrix block arising from the parameter-free mixed method was found to be better than the analogous HDG block for all ranges of the HDG stabilization parameter we considered.

**Outline.** We set up general notation in Sect. 2 and continue with a description of the variational framework used throughout the paper. Finite element spaces, a discrete Korn-like inequality, and resulting norm equivalences are introduced in Sect. 3. A list of interpolation operators into these spaces and their properties with references to literature can also be found there. In Sect. 4 we introduce and analyze the HDG method for the primal set of Eq. (1) and in Sect. 5 we do the same for the MCS method for the mixed set of eq. (2). Finally, in Sect. 6 we perform numerical experiments to illustrate and complement our theoretical findings.

## Notation and Weak Forms

By ${\mathbb {M}}$ we denote the vector space of real $3 \times 3$ matrices and by ${\mathbb {K}}$ we denote the vector space of $3 \times 3$ skew symmetric matrices, i.e., ${\mathbb {K}}= {\text {skw}}({\mathbb {M}})$, where ${\text {skw}}\tau = \frac{1}{2} (\tau - \tau ^T)$ for $\tau \in {\mathbb {M}}$. Further, let ${\mathbb {D}}= {\text {dev}}{({\mathbb {M}})}$. To indicate vector and matrix-valued functions on $\varOmega$, we include the range in the notation, thus while $L^2(\varOmega ) = L^2(\varOmega , {\mathbb {R}})$ denotes the space of square integrable and weakly differentiable ${\mathbb {R}}$-valued functions on $\varOmega$, the corresponding vector and matrix-valued function spaces are defined by $L^2(\varOmega , {\mathbb {R}}^3):= \left\{ u: \varOmega \rightarrow {\mathbb {R}}^3 \big | \;u_i \in L^2(\varOmega )\right\}$ and $L^2(\varOmega ,{\mathbb {M}}):= \left\{ \tau : \varOmega \rightarrow {{\mathbb {M}}} \big |\; \tau _{ij} \in L^2(\varOmega )\right\}$, respectively. For any ${\tilde{\varOmega }} \subseteq \varOmega$, we denote by $(\cdot ,\cdot )_{\tilde{\varOmega }}$ the inner product on $L^2({\tilde{\varOmega }})$ (or its vector- or matrix-valued versions). Similarly, we extend this notation and write $\Vert \cdot \Vert _{{\tilde{\varOmega }}}$ for the corresponding $L^2$-norm of a (scalar, vector, or matrix-valued) function on the domain ${\tilde{\varOmega }}$. In the case ${\tilde{\varOmega }} = \varOmega$ we will omit the subscript in the inner product, i.e. we have $(\cdot ,\cdot )_{\tilde{\varOmega }} = (\cdot ,\cdot )$ and we will use the notation $\Vert \cdot \Vert _0 = \Vert \cdot \Vert _\varOmega$.

In addition to the differential operators we have already used, $\nabla , \varepsilon , {\text {curl}}$, we understand ${\text {div}}\varPhi$ as either $\sum _{i=1}^3 \partial _i \varPhi _i$ for a vector-valued function $\varPhi$, or the row-wise divergence $\sum _{j=1}^3 \partial _j \tau _{ij}$ for a matrix-valued function $\tau$. In addition to the standard Sobolev spaces $H^m(\varOmega )$ for any $m\in {\mathbb {R}}$, we shall also use the well-known spaces $H({\text {div}},\varOmega ) = \{ v \in L^2(\varOmega , {\mathbb {R}}^3): {\text {div}}v \in L^2(\varOmega ) \}$ and $H({\text {curl}},\varOmega ) = \{ v \in L^2(\varOmega , {\mathbb {R}}^3): {\text {curl}}v \in L^2(\varOmega ,{\mathbb {R}}^3) \}$. We use $H^1_{0,B}(\varOmega )$, $H_{0,B}({\text {div}}, \varOmega )$ and $H_{0,B}({\text {curl}}, \varOmega )$, to denote the spaces of functions whose trace, normal trace and tangential trace respectively vanish on $\varGamma _B$, for $B \in \{ D, N \}$. The only somewhat nonstandard Sobolev space that we shall use is

5 $$\begin{aligned} H({\text {curl}}{\text {div}}, \varOmega ) := \{ \tau \in L^2(\varOmega ,{\mathbb {D}}): {\text {div}}\tau \in H_{0,D}({\text {div}}, \varOmega )^*\}, \end{aligned}$$

where $H_{0,D}({\text {div}}, \varOmega )^*$ is the dual space of $H_{0, D}({\text {div}}, \varOmega )$. In the case $\varGamma _D = \partial \varOmega$, as proved in [21], the dual of $H_{0, D}({\text {div}}, \varOmega )$ equals $H^{-1}({\text {curl}}, \varOmega )$, so the condition that ${\text {div}}\tau \in H_{0,D}({\text {div}}, \varOmega )^*$ in (5) is the same as requiring that ${\text {curl}}{\text {div}}\tau \in H^{-1}(\varOmega )$. This explains the presence of the operator “${\text {curl}}{\text {div}}$” in the name of the space in (5).

We denote by $\mathcal {T}$ a quasiuniform and shape regular triangulation of the domain $\varOmega$ into tetrahedra. Let *h* denote the maximum of the diameters of all elements in $\mathcal {T}$. Throughout this work we write $A \sim B$ when there exist two constants $c,C >0$ *independent of the mesh size has well as the viscosity *$\nu$ such that $cA \le B \le C A$. Similarly, we use the notation $A \lesssim B$ if there exists a similar constant *C* (independent of *h* and $\nu$) such that $A \le CB$. Henceforth we assume that $\nu$ is a constant. Due to quasiuniformity we have $h \sim \text {diam}(T)$ for any $T\in \mathcal {T}$. The set of element interfaces and boundaries is denoted by $\mathcal {F}$. This set is further split into facets on the Dirichlet boundary, $\mathcal {F}_D = \{ F \in \mathcal {F}: F \subset \varGamma _D\}$, facets on the Neumann boundary $\mathcal {F}_N = \{ F \in \mathcal {F}: F \subset \varGamma _N\}$ and facets in the interior $\mathcal {F}^{0} = \mathcal {F}{\setminus } ( \mathcal {F}_N \cup \mathcal {F}_D)$. Also let $\mathcal {F}_{0, D} = \mathcal {F}_0 \cup \mathcal {F}_D$.

For piecewise smooth functions *v* on the mesh, $\llbracket {{v}}\rrbracket$ and $\{ v \}$ are functions on $\mathcal {F}$ whose values on each interior facet equal the jump (defined up to a sign) of *v* and the mean of the values of *v* from adjacent elements. On boundary facets, they are both defined to be the trace of *v*. On each element boundary, and similarly on each facet on the global boundary we denote by *n* the outward unit normal vector. Then the normal and tangential trace of a smooth enough vector field *v* is given by

$$\begin{aligned} v_n = v\cdot n \quad \text {and} \quad v_t = v - v_n n. \end{aligned}$$

Accordingly, the normal trace is a scalar function and the tangential trace is a vector function. In a similar manner we introduce the normal-normal (*nn*) trace and the normal-tangential (*nt*) trace of a matrix valued function $\tau$ by

$$\begin{aligned} \tau _{nn} := \tau : n \otimes n = n^{{\text {T}}}\tau n \quad \text {and} \quad \tau _{nt} = (\tau n)_t. \end{aligned}$$

For any $\tilde{\varOmega } \subseteq \varOmega$, we denote by $P^k(\tilde{\varOmega }) = P^k(\tilde{\varOmega },{\mathbb {R}})$ the set of polynomials of degree at most *k*, restricted to $\tilde{\varOmega }$. Let $P^k(\tilde{\varOmega },{\mathbb {R}}^3)$ and $P^k(\tilde{\varOmega },{\mathbb {M}})$ denote the analogous vector- and matrix-valued versions whose components are in $P^k(\tilde{\varOmega })$. With respect to these spaces we then define $\varPi ^k_{\tilde{\varOmega }}$, the $L^2(\tilde{\varOmega })$-projection into the space $P^k(\tilde{\varOmega })$ or its vector- or matrix-valued versions. We omit subscript from $\varPi ^k_{\tilde{\varOmega }}$ if it is clear from context. For the space of functions the restrictions of which are in $P^k(T)$ for all $T\in \mathcal {T}$ we write simply $P^k(\mathcal {T})$. The analogous convention holds for $H^k(\mathcal {T}), L^2(\mathcal {F})$, etc.

The standard [18] variational formulation of (1) is to find $(u,p)\in H^1_{0,D}(\varOmega , {\mathbb {R}}^3)\times L^2(\varOmega )$ such that

6a $$\begin{aligned} \nu (\varepsilon (u),\varepsilon (v)) - ({\text {div}}v,p)&= (f,v){} & {} \quad \text { for all }v \in H^1_{0,D}(\varOmega , {\mathbb {R}}^3), \end{aligned}$$

6b $$\begin{aligned} - ({\text {div}}u,q)&= 0{} & {} \quad \text { for all }q \in L^2(\varOmega ). \end{aligned}$$

However our novel methods use $H({\text {div}})$-conforming spaces for the approximation of the velocity *u*. Another weak form where velocity is set in $H({\text {div}})$ was given in [20, 21, 24] using $\varSigma ^{{\text {sym}}}:= \{ \tau \in H({\text {curl}}{\text {div}}, \varOmega ): \;\tau = \tau ^{{\text {T}}}\}.$ It finds $(\sigma , u, p) \in \varSigma ^{{\text {sym}}} \times H_{0,D}({\text {div}}, \varOmega ) \times L^2(\varOmega )$ such that

7a $$\begin{aligned} (\nu ^{-1} \sigma , \tau ) + \langle {\text {div}}\tau , u\rangle _{{\text {div}}}&= 0{} & {} \quad \text { for all }\tau \in \varSigma ^{{\text {sym}}}, \end{aligned}$$

7b $$\begin{aligned} -\langle {\text {div}}\sigma , v\rangle _{{\text {div}}} - ({\text {div}}v, p)&= (f, v){} & {} \quad \text { for all }v \in H_{0,D}({\text {div}}, \varOmega ), \end{aligned}$$

7c $$\begin{aligned} -({\text {div}}u, q)&=0{} & {} \quad \text { for all }q \in L^2(\varOmega ), \end{aligned}$$

where $\varSigma ^{{\text {sym}}}:= \{ \tau \in H({\text {curl}}{\text {div}}, \varOmega ): \;\tau = \tau ^{{\text {T}}}\}.$ Here $\langle \cdot , \cdot \rangle _{{\text {div}}}$ denotes the duality pairing on $H_{0,D}({\text {div}}, \varOmega )^* \times H_{0,D}({\text {div}}, \varOmega )$. Note that since $\sigma \in L^2(\varOmega , {\mathbb {D}})$ we have ${{\text {tr}}(\sigma )} = 0$ which is motivated by (2a). In [24], a detailed well-posedness analysis of (7) was provided, but in this paper, (7) will serve merely to motivate the new mixed method of Sect. 5.

## The Finite Elements Used and Their Properties

In this preparatory section, we define the standard finite element spaces used to construct our methods, their natural interpolators, and a number of discrete norm equivalences revealing equivalent norms involving piecewise ${\varepsilon }(\cdot )$. Lemma 2 below will be used in the analysis of the HDG scheme while the analysis of the MCS scheme will additionally need Lemmas 3–4. We begin with the finite element spaces used in this paper:

8a $$\begin{aligned} V_h&:= \{v_h \in H_{0,D}({\text {div}},\varOmega ): v_h|_T \in P^1(T,{\mathbb {R}}^3)\}, \end{aligned}$$

8b $$\begin{aligned} \widehat{V}_h&:= \{ \widehat{v}_h \in L^2(\mathcal {F}, {\mathbb {R}}^3): \widehat{v}_h = 0 \text { on } \varGamma _D, \text { and for all } F \in \mathcal {F}, \nonumber \\& \widehat{v}_h|_F \in P^0(F,{\mathbb {R}}^3) \text { and } (\widehat{v}_h)_n|_F = 0 \}, \end{aligned}$$

8c $$\begin{aligned} W_h&:= \{ \eta _h \in H_{0,D}({\text {div}}, \varOmega ): \eta _h|_T \in P^0(T, {\mathbb {R}}^3) + x P^0(T,{\mathbb {R}})\text { for all }T \in \mathcal {T}\}, \end{aligned}$$

8d $$\begin{aligned} \varSigma _h&:= \{ \tau _h \in L^2(\varOmega , {\mathbb {D}}): \tau _h|_T \in P^1(T, {\mathbb {D}}), (\tau _h)_{nt}|_F \in P^0(F, n_F^\perp ) \}, \end{aligned}$$

8e $$\begin{aligned} Q_h&:= P^0(\mathcal {T}). \end{aligned}$$

Note that for any $\tau _h \in \varSigma _h$, on a facet *F*, $(\tau _h)_{nt}$ is a constant function on *F* taking values in $n_F^\perp$, where $n_F^\perp$ denotes the orthogonal complement of $n_F$, a unit normal of *F*. This is indicated by the notation $(\tau _h)_{nt} \in P^0(F,n_F^\perp )$ in (8d). Also any $\widehat{v}_h \in \widehat{V}_h$ is tangential and takes values in $n_F^\perp$ on each facet *F*. Note also that $V_h$, which equals $H_{0,D}({\text {div}},\varOmega ) \cap \mathcal {BDM}^1$ in the notation of §1, is the lowest order Brezzi-Douglas-Marini space while $W_h$ is the lowest order Raviart-Thomas space [3]. The space $\varSigma _h$ is a discontinuous version of the “*nt*-continuous” space introduced in [21], for which simple shape functions were exhibited there. All of these finite element spaces are obtained by mappings from a single reference finite element. (All these maps extend to curvilinear elements, although we restrict to affine equivalent elements in our analysis here.) The maps are compatible with the degrees of freedom of the spaces. (For $\varSigma _h$, the appropriate map is given in [21] and compatibility with degrees of freedom is proved in [21, Lemma 5.7], while for the other spaces, the mappings are standard.) In the case of $V_h$ and $W_h$, the maps are Piola maps which also preserve divergence-free subspaces.

### A Discrete Korn-Type Inequality

Korn inequalities for piecewise functions were given in [5, Theorem 3.1]. A further refinement was given in [31, Theorem 3.1]. To describe it, let ${\varPi ^{R}}$ denote the facet-wise $L^2$ projection onto ${R_{\scriptscriptstyle F}}:= \{t + \alpha ~ n\times x: t \in n^\perp ,~\alpha \in {\mathbb {R}}\}$, the space of tangential components (on a facet *F*) of the rigid displacements (or simply the space of two-dimensional rigid displacements on *F*). Let $H^1_{n, D}(\mathcal {T},{\mathbb {R}}^3):= \{ u: u \in H^1(T,{\mathbb {R}}^3)$ for all elements $T \in \mathcal {T}$ and ${\llbracket {{u}}\rrbracket }_n = 0$ on all facets $F \in \mathcal {F}_{0, D} \}$. A minor modification of the proof of [31, Theorem 3.1] shows that

9 $$\begin{aligned} \Vert \nabla u \Vert _\mathcal {T}^2&\;\lesssim \; \Vert \varepsilon (u) \Vert _\mathcal {T}^2+ h^{-1} \big \Vert {\varPi ^{R}}\llbracket {{u}}\rrbracket _t \big \Vert _{{\mathcal {F}_{0, D}}}^2 \quad \text { for all } u \in H^1_{n, D}(\mathcal {T}, {\mathbb {R}}^3). \end{aligned}$$

Here and throughout, we use $\Vert \cdot \Vert _\mathcal {T}^2$ to abbreviate $\sum _{T \in \mathcal {T}} \Vert \cdot \Vert _T^2$ with the understanding that any derivative operators in the argument of these norms are evaluated summand by summand, e.g., the gradient and $\varepsilon$ are evaluated element by element in (9). This notation is similarly extended to facets, so $\Vert \cdot \Vert _{{\mathcal {F}_{0, D}}}^2 =\sum _{F \in \mathcal {F}_{0, D}} \Vert \cdot \Vert _F^2$. Note how normal components are controlled in (9) through the space $H^1_{n, D}(\mathcal {T}, {\mathbb {R}}^3)$, while tangential components are controlled through the jumps $\llbracket {{u}}\rrbracket _t$. The next result shows that a part of the right hand side of (9) can be traded for a norm of the jump of $n \cdot {\text {curl}}u$ when *u* is in $V_h$.

#### Lemma 1

For all $$u_h \in V_h$$,

10 $$\begin{aligned} \begin{aligned} \Vert \varepsilon (u_h)\Vert _\mathcal {T}^2 + h^{-1}\big \Vert {\varPi ^{R}}\llbracket {{u_h}}\rrbracket _t\big \Vert _{{\mathcal {F}_{0, D}}}^2 \sim \; \Vert \varepsilon (u_h)\Vert _\mathcal {T}^2&+ h^{-1}\big \Vert \varPi ^0\llbracket {{u_h}}\rrbracket _t\big \Vert _{{\mathcal {F}_{0, D}}}^2 \\&+ h\big \Vert \llbracket {{{\text {curl}}u_h}}\rrbracket _n\big \Vert _{{\mathcal {F}_{0, D}}}^2. \end{aligned} \end{aligned}$$

#### Proof

By Pythagoras theorem,

$$\begin{aligned} \big \Vert {\varPi ^{R}}\llbracket {{u_h}}\rrbracket _t\big \Vert ^2_F = \big \Vert \varPi ^0\llbracket {{u_h}}\rrbracket _t\big \Vert ^2_F + \big \Vert ({\varPi ^{R}}-\varPi ^0)\llbracket {{u_h}}\rrbracket _t\big \Vert ^2_F. \end{aligned}$$

Hence (10) would follow once we prove that for all $F \in \mathcal {F}$ and all $u_h \in V_h$,

11 $$\begin{aligned} \begin{aligned} h\big \Vert \llbracket {{{\text {curl}}u_h}}\rrbracket _n&\big \Vert _F^2 + \sum _{T \in \mathcal {T}_F}\big \Vert \varepsilon (u_h)\big \Vert ^2_T \;\sim \; h^{-1}\big \Vert ({\varPi ^{R}}-\varPi ^0)\llbracket {{u_h}}\rrbracket _t\big \Vert _F^2 + \sum _{T\in \mathcal {T}_F}\big \Vert \varepsilon (u_h)\big \Vert ^2_T, \end{aligned} \end{aligned}$$

where $$\mathcal {T}_F = \{ T \in \mathcal {T}: F \subset \partial T\}$$.

To prove (11), first note that, restricted to every facet *F*, ${\varPi ^{R}}-\varPi ^0$ is the $L^2(F)$-orthogonal projection onto the one dimensional span of $r_F = n_F \times (x - x_F)$ where ${x_{\scriptscriptstyle F}}=\frac{1}{|F|}\int _Fx\mathop {{\textrm{d}} x}$ is the barycenter of *F*. Computing this one-dimensional projection, $({\varPi ^{R}}-\varPi ^0)\llbracket {{u_h}}\rrbracket _t\big |_{F} = ({r_{\scriptscriptstyle F}}, \llbracket {{u_h}}\rrbracket )_F \,r_F/\Vert {r_{\scriptscriptstyle F}}\Vert _F^2$. Therefore,

12 $$\begin{aligned} \big \Vert ({\varPi ^{R}}-\varPi ^0)\llbracket {{u_h}}\rrbracket _t\big \Vert _F = \frac{|({r_{\scriptscriptstyle F}}, \llbracket {{u_h}}\rrbracket )_F|}{\Vert {r_{\scriptscriptstyle F}}\Vert _F}. \end{aligned}$$

To simplify the numerator of the last term, let *w* equal $u_h|_T$ for some $T \in \mathcal {T}_F$. We claim that

13 $$\begin{aligned} (r_F, w)_F = (r_F, {\varepsilon }(w) (x - x_F))_F + \frac{1}{2} (|x - x_F|^2, n_F \cdot {\text {curl}}w)_F. \end{aligned}$$

To see why, recalling that *w* is linear in *T* (and hence in $F \subset \partial T$), for any $x \in F$,

$$\begin{aligned} w(x)&= w(x_F) + \nabla w\, ( x- x_F) \\&= w(x_F) + {\varepsilon }(w) (x- x_F) + \frac{1}{2} {\text {curl}}w \times (x-x_F), \end{aligned}$$

where we have used (3). Since $r_F$ is orthogonal to constants on *F*,

$$\begin{aligned} (r_F, w) = (r_F, {\varepsilon }(w) (x - x_F))_F + \frac{1}{2} (n_F \times (x - x_F), {\text {curl}}w \times (x - x_F))_F. \end{aligned}$$

Now, since $(x-{x_{\scriptscriptstyle F}})\perp n$ for any $x \in F$, using the identity $(a\times b) \cdot (c\times b) = |b|^2(a\cdot c) - (a\cdot b)(c\cdot b)$ to simplify the last term, we obtain (13).

The equivalence of (11) is a consequence of the identity

14 $$\begin{aligned} \big \Vert ({\varPi ^{R}}-\varPi ^0)\llbracket {{u_h}}\rrbracket _t\big \Vert _F = \frac{({r_{\scriptscriptstyle F}}, \llbracket {{{\varepsilon }(u_h)}}\rrbracket (x - x_F))_F}{\Vert {r_{\scriptscriptstyle F}}\Vert _F} + \frac{( |x - x_F|^2, \llbracket {{{\text {curl}}u_h}}\rrbracket _n )_F}{2\Vert {r_{\scriptscriptstyle F}}\Vert _F}, \end{aligned}$$

immediately obtained by combining (12) and (13). Indeed, by applying Cauchy–Schwarz inequality to the terms on the right hand side of (14), simple local scaling arguments give $h^{-1}\big \Vert ({\varPi ^{R}}-\varPi ^0)\llbracket {{u_h}}\rrbracket _t\big \Vert _F^2 \lesssim \sum _{T \in \mathcal {T}_F} \Vert {\varepsilon }(u_h) \Vert _T^2 + h\big \Vert \llbracket {{{\text {curl}}u_h}}\rrbracket _n\big \Vert _F^2,$ thus proving one side of equivalence in (11). To prove the other side, we begin by noting that ${\text {curl}}(u_h)$ is constant on each element, so

$$\begin{aligned} h^{1/2} \big \Vert \llbracket {{{\text {curl}}u_h}}\rrbracket _n\big \Vert _F&\lesssim h^{-1/2} \frac{( |x - x_F|^2, \llbracket {{{\text {curl}}u_h}}\rrbracket _n )_F}{2\Vert {r_{\scriptscriptstyle F}}\Vert _F} \\&= h^{-1/2} \left( \big \Vert ({\varPi ^{R}}-\varPi ^0)\llbracket {{u_h}}\rrbracket _t\big \Vert _F - \frac{({r_{\scriptscriptstyle F}}, \llbracket {{{\varepsilon }(u_h)}}\rrbracket (x - x_F))_F}{\Vert {r_{\scriptscriptstyle F}}\Vert _F} \right) \\&\lesssim h^{-1/2} \left\| ({\varPi ^{R}}-\varPi ^0)\llbracket {{u_h}}\rrbracket _t\right\| _F +\sum _{T \in \mathcal {T}_F}\Vert \varepsilon (u_h)\Vert _T, \end{aligned}$$

where we have used (14) and local scaling arguments again. Squaring both sides and applying Young’s inequality, (11) is proved. $\square$

### Norm Equivalences

The product space for the kinematic variables is given by $U_h:= V_h \times \widehat{V}_h \times W_h$. For the analysis we define the normswhere we have used $\Vert \cdot \Vert _{\partial \mathcal {T}}^2$ to abbreviate $\sum _{T \in \mathcal {T}} \Vert \cdot \Vert _{\partial T}^2$. We will shortly establish relationships between these norms (Lemma 4). That these are all norms on $U_h$ may not be immediately obvious, but follows from Lemma 2 below (where we critically use that $\widehat{u}_h$ is single valued on facets). As we shall see later,  is the natural norm to analyze the new HDG method in §4, while $\Vert \cdot \Vert _{\varepsilon }$ features in the analysis of the MCS method in §5. All the above norms involve the interface variable $\widehat{u}_h$, so they may be referred to as “HDG-type” norms. In contrast, “DG-type” norms were used in Subsection 3.1, where Lemma 1 and (9) imply

15 $$\begin{aligned} \Vert \nabla u_h \Vert _{\mathcal {T}} \;\lesssim \; \Vert \varepsilon (u_h)\Vert _\mathcal {T}^2 + h^{-1}\big \Vert \varPi ^0\llbracket {{u_h}}\rrbracket _t\big \Vert _{\mathcal {F}_{0, D}}^2 + h\big \Vert \llbracket {{{\text {curl}}u_h}}\rrbracket _n\big \Vert _{\mathcal {F}_{0, D}}^2. \end{aligned}$$

A similar discrete Korn-type inequality also holds for HDG-type norms, as seen in the next lemma.

#### Lemma 2

For all $(u_h, \widehat{u}_h, \omega _h)\in U_h$, we have the Korn-like inequality16The reverse inequality holds in the sense that for any $(u_h, \widehat{u}_h)\in V_h\times \widehat{V}_h$ there exists a $\omega _h\in W_h$ such that17

#### Proof

To prove (16), first note that on an interior facet $F = \partial T_+ \cap \partial T_- \in \mathcal {F}_0$ shared by two elements $T_\pm \in \mathcal {T}$, letting $u_h^\pm = u_h|_{T_\pm }$, since $\widehat{u}_h$ is single valued on *F*, we have $u_h^+ - u_h^- = (u_h^+ - \widehat{u}) - (u_h^- - \widehat{u})$. Moreover, on a facet $F \in \mathcal {F}_D$, $u_h|_F = (u_h - \widehat{u}_h)|_F$. Thus by triangle inequality,

18 $$\begin{aligned} \big \Vert \varPi ^0\llbracket {{u_h}}\rrbracket _t\big \Vert _{\mathcal {F}_{0, D}}^2&\le 2\Vert \varPi ^0(u_h - \widehat{u}_h)_t\Vert _{\partial \mathcal {T}}^2, \end{aligned}$$

where we have increased the right hand side to include facets on $\varGamma ^N$ also. Similarly, since the normal component of the given $\omega _h \in W_h$ is continuous across $F \in \mathcal {F}_0$ and zero on $F \in \mathcal {F}_D$,

19 $$\begin{aligned} \big \Vert \llbracket {{{\text {curl}}u_h}}\rrbracket _n\big \Vert _\mathcal {F}^2 \le 2 \left\| ({\text {curl}}u_h - \omega _h)_n\right\| _{\partial \mathcal {T}}^2. \end{aligned}$$

Using (18) and (19) in (15), we obtain the estimate (16).

To prove (17), consider a function $\omega _h\in W_h$ satisfying

$$\begin{aligned} \begin{aligned} n \cdot \omega _h&= n \cdot \{ {\text {curl}}u_h\}{} & {} \text { on } \partial T \setminus \varGamma ^D, \\ n \cdot \omega _h&= 0{} & {} \text { on } \partial T \cap \varGamma ^D, \end{aligned} \end{aligned}$$

on the boundary of every element $T \in \mathcal {T}$. Since ${\text {curl}}u_h$ is piecewise constant, by the well known degrees of freedom of the Raviart-Thomas space $W_h$, these conditions uniquely fix an $\omega _h \in W_h$. Then, $\Vert ({\text {curl}}u_h - \omega _h)_n\Vert _F$ equals zero for $F \in \mathcal {F}_N$, equals $\frac{1}{2} \big \Vert \llbracket {{{\text {curl}}u_h}}\rrbracket _n\big \Vert _F$ for $F \in \mathcal {F}_0$, and equals $\Vert ({\text {curl}}u_h)_n \Vert _F$ for $F \in \mathcal {F}_D$, so

$$\begin{aligned} \Vert ({\text {curl}}u_h - \omega _h)_n \Vert _{ \partial \mathcal {T}}^2 \lesssim \big \Vert \llbracket {{{\text {curl}}u_h}}\rrbracket _n \big \Vert _{\mathcal {F}_{0, D}}^2. \end{aligned}$$

Therefore, for this choice of $\omega _h$, we haveBy a local scaling argument $h \big \Vert \llbracket {{{\text {curl}}u_h}}\rrbracket _n\big \Vert _{\mathcal {F}}^2 \lesssim \Vert {\text {curl}}u_h \Vert _\mathcal {T}^2$. Using this in the above inequality and recalling that $\Vert \nabla u_h \Vert _\mathcal {T}^2 = \Vert {\varepsilon }(u_h) \Vert _\mathcal {T}^2 + \Vert \kappa ( {\text {curl}}u_h) \Vert _\mathcal {T}^2$, we complete the proof of (17). $\square$

#### Lemma 3

For any $u_h\in V_h$, $\omega _h\in W_h$, and $T \in \mathcal {T}$,

20a $$\begin{aligned}&\Vert {\text {curl}}u_h - \omega _h\Vert _T^2 \sim ~ h \Vert ({\text {curl}}u_h - \omega _h)_n\Vert _{\partial T}^2, \end{aligned}$$

20b $$\begin{aligned}&\Vert {\text {curl}}\kappa (\omega _h) \Vert _T \sim ~ | \kappa (\omega _h) |_{H^1(T)}^2 \sim \Vert {\text {div}}\omega _h \Vert _T, \end{aligned}$$

20c $$\begin{aligned}&\begin{aligned}&\Vert \varepsilon (u_h) \Vert ^2_T + \Vert {\text {curl}}u_h - \omega _h\Vert _T^2 \sim ~ \Vert {\text {dev}}\nabla u_h - \varPi ^0 \kappa (\omega _h) \Vert _T^2 \\&+ ~ h^2 \Vert {\text {div}}\omega _h \Vert _T^2 + \Vert {\text {div}}u_h \Vert _T^2. \end{aligned} \end{aligned}$$

#### Proof

The first equivalence follows by standard scaling arguments (by equivalence of norms in the lowest order Raviart-Thomas space). Equivalence (20b) also follows by local scaling arguments and [20, eq. (4.14)]. We continue on to prove (20c). Applying the Pythagoras theorem twice,

21 $$\begin{aligned} \Vert \varepsilon (u_h) \Vert _T^2 + \frac{1}{2} \Vert {\text {curl}}u_h - \omega _h\Vert _T^2&= \Vert \nabla u_h - \kappa (\omega _h)\Vert _T^2 \nonumber \\&= \Vert {\text {dev}}\nabla u_h - \kappa (\omega _h)\Vert _T^2 + \frac{1}{3} \Vert {\text {div}}u_h\Vert _T^2. \end{aligned}$$

We also have, due to (20b),

22 $$\begin{aligned} \begin{aligned} h^2 \Vert {\text {div}}\omega _h \Vert ^2_T \sim h^2 \Vert {\text {curl}}(\kappa (\omega _h)) \Vert ^2_T&~= h^2\big \Vert {\text {curl}}\big (\kappa (\omega _h-{\text {curl}}u_h) \big )\big \Vert ^2_T \\&~\lesssim \Vert \omega _h - {\text {curl}}u_h \Vert _T^2. \end{aligned} \end{aligned}$$

Here we have used an inverse inequality and the observation that derivatives of ${\text {curl}}u_h \in P^0(T)$ vanish. Combining (21), (22) and the continuity of the $L^2$ projection, we conclude that the right side of (20c) can be bounded by the left side.

For the reverse inequality,

23 $$\begin{aligned} \Vert {\text {dev}}\nabla u_h - \kappa (\omega _h)\Vert ^2_T&= \Vert \varPi ^0( {\text {dev}}\nabla u_h - \kappa (\omega _h))\Vert _T^2 + \Vert ({\text {I}}- \varPi ^0) \kappa (\omega _h)\Vert _T^2 \nonumber \\&\lesssim \Vert {\text {dev}}\nabla u_h - \varPi ^0 \kappa (\omega _h)\Vert _T^2 + h^2 | \kappa (\omega _h)|_{H^1(T)}^2 \nonumber \\&\sim \Vert {\text {dev}}\nabla u_h - \varPi ^0 \kappa (\omega _h)\Vert _T^2 + h^2 \Vert {\text {div}}\omega _h\Vert _T^2. \end{aligned}$$

Here, we used that ${\text {dev}}\nabla u_h \in P^0(T)$, a standard approximation estimate for the $L^2$ projection, followed by (20b). The proof is then concluded using (21). $\square$

#### Lemma 4

For all $$(u_h, \widehat{u}_h, \omega _h)\in U_h$$,

#### Proof

This is a direct consequence of Lemma 3. $\square$

### Interpolation Operators

In subsequent sections we will require the interpolation operators into the spaces in (8a)-(8e), denoted by

$$\begin{aligned} I_V: H^1_{n, D}(\mathcal {T})\rightarrow V_h,\quad I_W: H^1_{n, D}(\mathcal {T})\rightarrow W_h,\quad I_{\hat{V}}: L^2(\mathcal {F}, {\mathbb {R}}^3)\rightarrow \widehat{V}_h, \quad I_\varSigma : \varSigma \rightarrow \varSigma _h, \end{aligned}$$

where $\varSigma = \{\tau \in H^{1}(\mathcal {T}, {\mathbb {D}}):\llbracket {{\tau }}\rrbracket _{nt} = 0\}$. Of course, the natural interpolation for $Q_h$, denoted by $I_Q: L^2(\varOmega ) \rightarrow Q_h$, is simply the $L^2$-orthogonal projection. The definitions and properties of the remaining interpolants are summarized in this subsection.

An $H({\text {div}})$-interpolation into $V_h$, denoted by $I_V: H^1_{n, D}(\mathcal {T})\rightarrow V_h$, is defined using the standard degrees of freedom (see e.g., [3, Proposition 2.3.2]):

24 $$\begin{aligned} \quad \int _{F} (u - I_Vu)_n \,q\, \mathop {{\textrm{d}} s}= 0 \quad \text {for all } q\in P^1(F) \text { and } F\in \mathcal {F}. \end{aligned}$$

A well-known consequence of (24) is that

25 $$\begin{aligned} {\text {div}}(I_Vu) = I_Q{\text {div}}u, \end{aligned}$$

for all *u* in the domain of $I_V$. The interpolant $I_W: H^1_{n, D}(\mathcal {T})\rightarrow W_h$, defined by $((\omega - I_W\omega )_n, q)_F = 0$ for all $q\in P^0(F)$ and all $F\in \mathcal {F}$, is also standard. The interpolation operator for the stress space $I_\varSigma : \varSigma \rightarrow \varSigma _h$, borrowed from [21], is defined by

26 $$\begin{aligned}&\int _F(I_\varSigma \sigma - \sigma )_{nt} \cdot q \mathop {{\textrm{d}} s}= 0,{} & {} \text { for all }q\in P^0(F, {\mathbb {R}}^3) \text { with }q_n=0,~\text { for all }F\in \mathcal {F}, \end{aligned}$$

27 $$\begin{aligned}&\int _T(I_\varSigma \sigma - \sigma ) : q \mathop {{\textrm{d}} x}= 0,{} & {} \text { for all }q\in P^0(T, {\mathbb {D}}),~\text { for all }T\in \mathcal {T}. \end{aligned}$$

Finally, the tangential $$L^2$$-projection on facets, $$I_{\hat{V}}: L^2(\mathcal {F}, {\mathbb {R}}^3)\rightarrow \widehat{V}_h$$ is defined as usual by $$((\widehat{u} - I_{\hat{V}}\widehat{u})_t, q)_F = 0$$ for all $$q\in P^0(F, {\mathbb {R}}^3)$$ with $$ q_n=0$$ on all $$F\in \mathcal {F}$$.

To note the salient approximation properties of these interpolants, first observe that for a $u \in H^1(\varOmega , {\mathbb {R}}^3) \cap H^2(\mathcal {T})$, we have ${\text {curl}}(u) \in H^1_{n, D}(\mathcal {T})$. Hence $(I_Vu, I_{\hat{V}}u_t, I_W{\text {curl}}(u))$ is in $U_h$ and using standard scaling arguments and the Bramble-Hilbert lemma, we get28Also recall that [21, Theorem 5.8] implies that for all $\sigma \in \varSigma$,

29 $$\begin{aligned} \Vert \sigma - I_\varSigma \sigma \Vert _0^2 + h \left\| (\sigma - I_\varSigma \sigma )_{nt} \right\| _{\partial \mathcal {T}}^2&\lesssim \; h^2 \Vert \sigma \Vert ^2_{H^1(\mathcal {T})}. \end{aligned}$$

## An $H({\text {div}})$-Conforming Velocity–Vorticity HDG Scheme

### Derivation of the HDG Method

To derive our new HDG scheme for (6), let *u*, *p* be a sufficiently smooth exact solution of (1). (A sufficient smoothness condition is quantified in Lemma 5 below.) Let $v_h \in V_h$. Then, multiplying (1a) by $v_h$ and integrating by parts on each element,

30a $$\begin{aligned} \begin{aligned} (f, v_h) = (-{\text {div}}(\nu \varepsilon (u)) + \nabla p, v_h)&= -(p, {\text {div}}v_h) + \sum \limits _{T \in \mathcal {T}} \int _T \nu \varepsilon (u): \varepsilon (v_h) \mathop {{\textrm{d}} x}\\&\quad + \sum \limits _{T \in \mathcal {T}} \int _{\partial T \setminus \varGamma _N} (p - \nu \varepsilon (u))n \cdot v_h \mathop {{\textrm{d}} s}, \end{aligned} \end{aligned}$$

where we used the symmetry of $\varepsilon (u)$ and the boundary condition (1d). Since *p* is smooth, $\llbracket {{v_h}}\rrbracket _n=0$ on $\mathcal {F}_{0, D}$,

30b $$\begin{aligned} 0 = -\sum \limits _{T \in \mathcal {T}} \int _{\partial T \setminus \varGamma _N} p n \cdot v_h \mathop {{\textrm{d}} s}. \end{aligned}$$

Let $\widehat{v}_h \in \widehat{V}_h$. Since $\widehat{v}_h$ is single-valued on all facets, $\widehat{v}_h=0$ on $\varGamma _D$ (see (8b)), and $\varepsilon (u)$ is continuous across interior facets,

30c $$\begin{aligned} 0 = \sum \limits _{T \in \mathcal {T}} \int _{\partial T \setminus \varGamma _N } \nu {\varepsilon }(u) n \cdot \widehat{v}_h \mathop {{\textrm{d}} s}. \end{aligned}$$

Adding (30a)–(30c),

$$\begin{aligned} \begin{aligned} (f, v_h)&= -(p, {\text {div}}v_h) + \sum \limits _{T \in \mathcal {T}} \int _T \nu \varepsilon (u): \varepsilon (v_h) \mathop {{\textrm{d}} x}+ \int _{\partial T \setminus \varGamma _N} \nu \varepsilon (u)n \cdot ( \widehat{v}_h - v_h) \mathop {{\textrm{d}} s}, \end{aligned} \end{aligned}$$

Since $(\widehat{v}_h)_t = \widehat{v}_h$, $\llbracket {{v_h}}\rrbracket _n=0$ on $\mathcal {F}_{0, D}$ and ${\varepsilon }(u)$ is smooth, we may replace $( \widehat{v}_h - v_h)$ by its tangential component $( \widehat{v}_h - v_h)_t$ in the last term above. Furthermore, on $\varGamma _N$, we have ${\varepsilon }(u) n \cdot (\widehat{v}_h - v_h)_t = {\varepsilon }(u)_{nt} \cdot (\widehat{v}_h - v_h)_t = 0$ since the tangential part of (1d) shows that $\varepsilon (u)_{nt}=0$ on $\varGamma _N$. Hence we may also replace $\partial T {\setminus } \varGamma _N$ by $\partial T$ in the last term. Thus,

31a $$\begin{aligned} \begin{aligned} (f, v_h)&= -(p, {\text {div}}v_h) + \sum \limits _{T \in \mathcal {T}} \int _T \nu \varepsilon (u): \varepsilon (v_h) \mathop {{\textrm{d}} x}+ \int _{\partial T} \nu \varepsilon (u)n \cdot ( \widehat{v}_h - v_h)_t \mathop {{\textrm{d}} s}. \end{aligned} \end{aligned}$$

Next, let $\omega = {\text {curl}}(u)$ and $\widehat{u} = u_t$ on each element boundary $\partial T$. Then, obviously,

31b $$\begin{aligned} 0&= \sum \limits _{T \in \mathcal {T}}\int _{\partial T} \nu \varepsilon (v_h)n \cdot (\widehat{u} - u)_t \mathop {{\textrm{d}} s}+ \sum \limits _{T \in \mathcal {T}}\frac{\nu \alpha }{h} \int _{\partial T} \varPi ^0(\widehat{u} - u)_t \cdot \varPi ^0(\widehat{v}_h - v_h)_t \mathop {{\textrm{d}} s}, \end{aligned}$$

31c $$\begin{aligned} 0&= \sum \limits _{T \in \mathcal {T}}h \int _{\partial T} \nu ({\text {curl}}u - \omega )_n ({\text {curl}}v_h - \eta _h)_n \mathop {{\textrm{d}} x}, \end{aligned}$$

for any test function $\eta _h \in W_h$ and constant $\alpha > 0$, i.e., if $u, \widehat{u}$ and $\omega$ are replaced by $u_h, \widehat{u}_h$ and $\omega _h$, respectively, then the terms on the right are consistent terms.

Adding the equations (31a)–(31c), we obtain

32a $$\begin{aligned} \nu a^{{\text {hdg}}}(u, \widehat{u}, \omega ; v_h, \widehat{v}_h, \eta _h) - ({\text {div}}v_h, p) = (f, v_h), \end{aligned}$$

where

$$\begin{aligned} a^{{\text {hdg}}}&(z, \widehat{z}, \theta ;\, v_h, \widehat{v}_h, \eta _h)\, := \left( \varepsilon (z), \varepsilon (v_h)\right) _{\mathcal {T}} \\&+ \left( \varepsilon (z) n, (\widehat{v}_h - v_h)_t\right) _{\partial \mathcal {T}} + \left( (\widehat{z} - z)_t, \varepsilon (v_h) n\right) _{\partial \mathcal {T}} \\&+ \frac{\alpha }{h} \left( \varPi ^0(\widehat{z} - z)_t, \varPi ^0(\widehat{v}_h - v_h)_t\right) _{\partial \mathcal {T}} + h \left( ({\text {curl}}z - \theta )_n, ({\text {curl}}v_h - \eta _h)_n\right) _{\partial \mathcal {T}}. \end{aligned}$$

Here and throughout, $(\cdot , \cdot )_\mathcal {T}= \sum _{T \in \mathcal {T}} (\cdot , \cdot )_T$ and $(\cdot , \cdot )_{\partial \mathcal {T}} = \sum _{T \in \mathcal {T}} (\cdot , \cdot )_{\partial T}$, extending our prior analogous norm notation to inner products. Of course, from (1b), we also have

32b $$\begin{aligned} ({\text {div}}u, q_h) = 0, \end{aligned}$$

for all $q_h \in Q_h$. Equations (32a)–(32b), after replacing $(u, \widehat{u}, \omega )$ by $(u_h, \widehat{u}_h, \omega _h)$, yield the following discrete formulation: find $(u_h, \widehat{u}_h, \omega _h) \in U_h$ and $p_h \in Q_h$ such that

33a $$\begin{aligned} \nu \,a^{{\text {hdg}}}(u_h,\widehat{u}_h, \omega _h;v_h, \widehat{v}_h, \eta _h) - (p_h, {\text {div}}v_h)&= (f,v_h), \end{aligned}$$

33b $$\begin{aligned} -({\text {div}}u_h, q_h)&= 0, \end{aligned}$$

for all $(v_h, \widehat{v}_h, \eta _h) \in U_h$ and $q_h \in Q_h$. Note that this method enforces $\varPi ^0(u_h)_t=0$ on $\varGamma _D$ as a consequence of how the last term of (31b) manifest in the method. Due to the Dirichlet conditions built into $W_h$ (see (8c)) the method also penalizes $\Vert \varPi ^0{\text {curl}}(u_h)_n\Vert _{\varGamma _D}$ through the manifestation of the consistent term (31c) in the method. System (33) may be thought of as a nonconforming HDG discretization of the standard weak form (6).

Note that $a^{{\text {hdg}}}(u, \widehat{u}, \omega ; v_h, \widehat{v}_h, \eta _h)$ is well defined for any $(v_h, \widehat{v}_h, \eta _h) \in U_h$ and any $(u, \widehat{u}, \omega ) \in U_{{\text {reg}}}$, where

34a $$\begin{aligned} U_{{\text {reg}}}&:= (H_{0,D}^1(\varOmega ) \cap H^2(\mathcal {T})) \times L^2(\mathcal {F}) \times H^1_{n, D}(\mathcal {T}), \end{aligned}$$

34b $$\begin{aligned} Q_{{\text {reg}}}&:= Q \cap H^1(\mathcal {T}). \end{aligned}$$

#### Lemma 5

(Consistency of the HDG method) Suppose the exact solution (*u*, *p*) of (6) is regular enough so that *u*, together with $\widehat{u} = u_t$ on facets and $\omega = {\text {curl}}u$, satisfies $(u, \widehat{u}, \omega ) \in U_{{\text {reg}}}$ and suppose $p \in Q_{{\text {reg}}}$. Then any $((u_h, \widehat{u}_h, \omega _h),p_h) \in U_h \times Q_h$ solving (33) satisfies

$$\begin{aligned} \nu a^{{\text {hdg}}}(u-u_h, u_t - \widehat{u}_h, \omega - \omega _h; v_h, \widehat{v}_h, \eta _h) - ({\text {div}}v_h,p - p_h) = 0, \end{aligned}$$

for all $$(v_h, \widehat{v}_h, \eta _h) \in U_h$$.

#### Proof

This follows by subtracting (33) from (32). $\square$

### Pressure Robust Error Analysis of the HDG Scheme

We follow the usual mixed method approach and proceed to combine continuity and coercivity of $a^{{\text {hdg}}}$ with a discrete Stokes inf-sup condition, or the LBB [3] estimate. The latter implies the stability of (33), which also implies its unique solvability. We begin by noting that by local scaling arguments, there is a mesh-independent $c_1$ such that

35 $$\begin{aligned} h \Vert \varepsilon (v_h) \Vert ^2_{\partial T} \le c_1 \Vert \varepsilon (v_h)\Vert ^2_T, \qquad v_h \in V_h, \quad T \in \mathcal {T}, \end{aligned}$$

since ${\varepsilon }(v_h)$ is constant on *T*. For the same reason, $\varPi ^0$ may be introduced into the second and third terms in the definition of $a^{{\text {hdg}}}(u_h,\widehat{u}_h, \omega _h;v_h, \widehat{v}_h, \omega _h)$, e.g.,

36 $$\begin{aligned} \left( {\varepsilon }(u_h)n, (\widehat{v}_h - v)_t \right) _{\partial \mathcal {T}} = \left( {\varepsilon }(u_h)n, \varPi ^0(\widehat{v}_h - v)_t \right) _{\partial \mathcal {T}}. \end{aligned}$$

Let 

#### Lemma 6

(Continuity of of $a^{{\text {hdg}}}$) For any $(u, \widehat{u}, \omega ) \in U_{{\text {reg}}}$, $(u_h,\widehat{u}_h, \omega _h) \in U_h,$ $(v_h, \widehat{v}_h, \omega _h) \in U_h$ and $q_h \in Q_h$,373839

#### Proof

Inequality (37) follows from Cauchy–Schwarz inequality, while (38) follows by additionally employing (35) and (36). The estimate (39) is a consequence of $\frac{1}{3} \Vert {\text {div}}u_h \Vert ^2_T = \Vert {\varepsilon }(u_h) \Vert _T^2 - \Vert {\text {dev}}{\varepsilon }(u_h) \Vert ^2_T \le \Vert {\varepsilon }(u_h)\Vert _T^2$. $\square$

#### Lemma 7

(Coercivity of $a^{{\text {hdg}}}$) There is a mesh-independent $\alpha _0>0$ such that for all $\alpha >\alpha _0$ and all $(u_h, \widehat{u}_h, \omega _h)\in U_h$,40

#### Proof

By (36) and Young’s inequality with any $\beta >0$,

$$\begin{aligned} a^{{\text {hdg}}}(u_h,\widehat{u}_h, \omega _h;u_h, \widehat{u}_h, \omega _h)&\ge \Vert \varepsilon (u_h) \Vert _\mathcal {T}^2 - \Big ( \beta h\Vert {\varepsilon }(u_h) n \Vert _{\partial \mathcal {T}}^2 + \frac{1}{\beta h} \Vert \varPi ^0 (u_h - \widehat{u}_h)_t\Vert ^2_{\partial \mathcal {T}} \Big ) \\&\quad + \alpha h^{-1} \Vert \varPi ^0 (u_h - \widehat{u}_h)_t\Vert ^2_{\partial \mathcal {T}} + h \Vert ({\text {curl}}u_h - \omega _h)_n\Vert _{\partial \mathcal {T}}^2. \end{aligned}$$

Hence using (35), and choosing, say $\beta = 1/(2c_1)$ and $\alpha = 2/\beta$, (40) follows. $\square$

#### Lemma 8

(LBB condition for the HDG method) For any $p_h\in Q_h$ there exists a $(v_h, \widehat{v}_h, \eta _h)\in U_h$ with  and ${\text {div}}v_h = p_h$. Consequently,41

#### Proof

By classical results [18], there exists a $u \in H^1(\varOmega )$ such that

42 $$\begin{aligned} {\text {div}}v = p_h, \qquad \Vert v \Vert _{H^1(\varOmega )} \lesssim \Vert p_h \Vert _0. \end{aligned}$$

Put $v_h = I_Vv$ and $\widehat{v}_h = I_{\hat{V}}v$ on each facet. Then, (42) and (25) imply ${\text {div}}v_h = {\text {div}}( I_Vv) = I_Q{\text {div}}v = p_h$. Moreover, (as alluded to in [29]) it is easy to show that

43 $$\begin{aligned} \left\| v_h, \widehat{v}_h \right\| _\nabla \lesssim \Vert v \Vert _{H^1(\varOmega )}. \end{aligned}$$

Choose $\eta _h\in W_h$ as in (17) of Lemma 2. Then, by (42)–(43),concluding the proof. $\square$

#### Theorem 1

(Error estimates for the HDG method) Let $u, \widehat{u}, \omega , p$ denote the exact solution that satisfies the regularity assumption of Lemma 5 and let $((u_h, \widehat{u}_h, \omega _h),p_h) \in U_h \times Q_h$ be the discrete solution of (33). Then the errors in $u_h, \widehat{u}_h, \omega _h$ can be bounded independently of the pressure error by44Furthermore, the pressure error satisfies

45 $$\begin{aligned} \nu ^{-1} \Vert p - p_h\Vert _0 \lesssim h ( \Vert u\Vert _{H^2(\mathcal {T})} + \nu ^{-1} \Vert p \Vert _{H^1(\mathcal {T})}). \end{aligned}$$

#### Proof

Let $E= (u - u_h, \widehat{u} - \widehat{u}_h, \omega - \omega _h)$ and $E_h = (I_Vu - u_h, I_{\hat{V}}\widehat{u} - \widehat{u}_h, I_W\omega - \omega _h)$. Then $\mathcal {E}= E - E_h$ represents the interpolation errors. Since $E_h \in U_h$,By (25), ${\text {div}}(I_Vu) = I_Q{\text {div}}u = 0$. Moreover, by (33b), ${\text {div}}u_h = 0$. Hence46by Lemma 6. Now we claim that47To see this, first note that local scaling arguments give

48 $$\begin{aligned} h^{-1} \Vert (I - \varPi ^0) v_h \Vert ^2_{\partial \mathcal {T}} \lesssim \Vert \nabla v_h \Vert ^2_\mathcal {T}, \end{aligned}$$

for any $v_h \in V_h$. Then, letting $E_h^u = I_Vu - u_h$, $E_h^{\widehat{u}} = I_{\hat{V}}u - \widehat{u}_h$, note that on each facet, $(I - \varPi ^0) \left( E_h^u - E_h^{\widehat{u}} \right) = (I - \varPi ^0)E_h^u$. Hence the extra terms in  that are not in  can be bounded by applying (48) and (35) with $v_h = E_h^u$ to getby Lemma 2. This proves (47). Using (47) in (46), we conclude that . Combining with triangle inequality,49where we have applied (28) in the last step. This proves (44).

For the pressure estimate, we begin with triangle inequality and Lemma 8:To bound the numerator of the supremum, we use Lemma 5:Hence the already proved estimate (49), together with the standard $L^2$ projection error estimates finish the proof of (45). $\square$

## An MCS Formulation with $H({\text {div}})$-Conforming Vorticity

In this section we derive a new mixed method for the approximation of (2), motivated by the weak formulation (7). Let $\sigma _h \in \varSigma _h$ and $(v_h,\widehat{v}_h, \eta _h) \in U_h$. Defining

50 $$\begin{aligned} \begin{aligned} \langle {\text {div}}\sigma _h ; \, v_h,\widehat{v}_h, \eta _h \rangle _{U_h} :=&\; ({\text {div}}\sigma _h, v_h)_\mathcal {T}- \left( (\sigma _h)_{nn}, (v_h)_n \right) _{\partial \mathcal {T}} \\&-\left( (\sigma _h)_{nt}, (\widehat{v}_h)_t\right) _{\partial \mathcal {T}} + \left( \sigma _h, \kappa (\eta _h)\right) _\mathcal {T}, \end{aligned} \end{aligned}$$

consider the terms on the right. When $(\sigma _h)_{nt}$ is continuous across element interfaces, the first two terms together realizes the duality pairing introduced in Sect. 2, namely $\langle {\text {div}}\sigma , v_h\rangle _{{\text {div}}}$, per [20, Theorem 3.1]. The third term is used to impose the *nt*-continuity of the viscous stress (and prior works [20, 21, 24] provided enough rationale to employ *nt*-continuous finite elements for viscous stresses). Note, that a similar nt-continuous approximation of the gradient (but not the physical viscous stresses $\varepsilon (u)$) was also already considered in [17]. Due to the Dirichlet conditions built into $\widehat{V}_h$ on $\varGamma _D$ (see (8b)), this term is comprised only of integrals over facets in the interior and on $\varGamma _N$, with the latter enforcing $\sigma _{nt}=0$ in $\varGamma _N$ as demanded by (2f). Finally, the last term above is used to weakly incorporate the symmetry constraint (2c). This technique of imposing symmetry weakly is widely used in finite elements for linear elasticity [1, 2, 4, 6, 15, 19, 34].

Viewing (7) in terms of $\langle {\text {div}}\cdot , \cdot \rangle _{U_h}$, we are led to the following mixed method: find $(u_h, \widehat{u}_h, \omega _h) \in U_h$ and $(\sigma _h,p_h) \in (\varSigma _h \times Q_h)$ satisfying

51a $$\begin{aligned} {\nu }^{-1}(\sigma _h, \tau _h) + \langle {\text {div}}\tau _h; \, u_h,\widehat{u}_h, \omega _h \rangle _{U_h}&= 0, \end{aligned}$$

51b $$\begin{aligned} -\langle {\text {div}}\sigma _h; \,v_h,\widehat{v}_h, \eta _h \rangle _{U_h} -({\text {div}}v_h, p_h) + c(\omega _h,\eta _h)&= (f,v_h), \end{aligned}$$

51c $$\begin{aligned} -({\text {div}}u_h, q_h)&= 0, \end{aligned}$$

for all $\tau _h \in \varSigma _h$, $(v_h, \widehat{v}_h, \eta _h) \in U_h$, and $q_h \in Q_h$, with the stabilizing bilinear form $c(\omega _h,\eta _h):= \nu h^2 ({\text {div}}\omega _h, {\text {div}}\eta _h)_\varOmega$. Note that since $\omega _h$ approximates the vorticity $\omega = {\text {curl}}(u)$, we have ${\text {div}}\omega =0$, so $c(\cdot , \cdot )$ is a consistent addition. Although the formulation (51) is very similar to the formulations from [20, 21], note the following differences. First, while the *nt*-continuity of viscous stresses was built into the spaces in [20, 21], now it is incorporated as an equation of the method by the well-known hybridization technique. Second, although we use the same local stress finite element space as in [21], we use the weak symmetric setting from [20]. In the latter, the Lagrange multiplier for the weak symmetry constraint was given by an element-wise discontinuous approximation, whereas here it is in the div-conforming $W_h$.

### Stability of the MCS Method

From the terms in (51), we anticipate that the norms $\Vert \cdot \Vert _{U_h}$ and $\Vert \cdot \Vert _\varepsilon$ are more natural for the analysis of the MCS method (in contrast to the HDG method). The latter appears in the next lemma.

#### Lemma 9

(Continuity of MCS formulation) The bilinear forms in (51) are continuous in the sense that for all $\sigma _h,\tau _h \in \varSigma _h$, $p_h \in Q_h$, $\eta _h \in W_h$ and $(u_h,\widehat{u}_h, \omega _h) \in U_h$, in addition to the obvious estimates

$$\begin{aligned} \nu ^{-1}(\sigma _h,\tau _h) \lesssim {{\nu }}^{-1/2} \Vert \sigma _h \Vert _0\, {{\nu }}^{-1/2} \Vert \tau _h \Vert _0, \quad \text {and} \quad c(\omega _h, \eta _h) \lesssim \nu h^2 \Vert {\text {div}}\omega _h \Vert _0\, \Vert {\text {div}}\eta _h\Vert _0, \end{aligned}$$

the following estimates hold:

52a $$\begin{aligned} ({\text {div}}u_h, p_h)&\lesssim \Vert u_h,\widehat{u}_h, \omega _h\Vert _{\varepsilon } \,\Vert p_h \Vert _{0}, \end{aligned}$$

52b $$\begin{aligned} \langle {\text {div}}\sigma _h; \, u_h,\widehat{u}_h, \omega _h \rangle _{U_h}&\lesssim \Vert \sigma _h \Vert _0 \,\Vert u_h,\widehat{u}_h, \omega _h \Vert _{\varepsilon }. \end{aligned}$$

#### Proof

Inequality (52a) is proved just like (39). To prove (52b), let us first note an equivalent and more compact form of $\langle {\text {div}}\sigma _h;\, v_h,\widehat{v}_h, \eta _h \rangle _{U_h}$ obtained by integrating (50) by parts (see e.g, [20, eq. (3.11)]), namely

53 $$\begin{aligned} \langle {\text {div}}\sigma _h;\,&v_h,\widehat{v}_h, \eta _h \rangle _{U_h} = -(\sigma _h, \nabla v_h - \kappa (\eta _h))_\mathcal {T}+ ( (\sigma _h)_{nt}, (v_h - \widehat{v}_h)_t)_{\partial \mathcal {T}}. \end{aligned}$$

Using (53), the fact that $\sigma _h$ is trace-free, the Cauchy–Schwarz inequality, and the following estimate (which follows by a local scaling argument using a specific mapping mentioned in the beginning of §3),

54 $$\begin{aligned} h^{1/2} \Vert (\sigma _h)_{nt} \Vert _{\partial \mathcal {T}} \lesssim \Vert \sigma _h \Vert _\mathcal {T}, \end{aligned}$$

we get

$$\begin{aligned} \langle {\text {div}}\sigma _h; \, u_h,\widehat{u}_h, \omega _h \rangle _{U_h}&\lesssim \Vert \sigma _h \Vert _0 \left( \Vert {\text {dev}}\nabla u_h - \kappa (\omega _h) \Vert ^2_\mathcal {T}+ h^{-1} \Vert \varPi ^0( u_h - \widehat{u}_h\Vert _{\partial \mathcal {T}}^2 \right) ^{1/2} \\&\lesssim \Vert \sigma _h\Vert _0 \left( \Vert u_h,\widehat{u}_h, \omega _h\Vert _{U_h}^2 + h^2 \Vert {\text {div}}\omega _h \Vert _\mathcal {T}^2\right) ^{1/2}, \end{aligned}$$

where the last inequality is due to the same argument as in (23). Thus (52b) follows from Lemma 4. $\square$

#### Lemma 10

For any $(u_h, \widehat{u}_h, \omega _h) \in U_h$ there exists a $(\tau _h,q_h) \in \varSigma _h \times Q_h$ satisfying

55 $$\begin{aligned} \Vert \tau _h\Vert _0 + \Vert q_h \Vert _0&\lesssim \Vert u_h, \widehat{u}_h, \omega _h\Vert _{U_h} + \Vert {\text {div}}u_h \Vert _0, \end{aligned}$$

56 $$\begin{aligned} \langle {\text {div}}\tau _h; \, u_h,\widehat{u}_h, \omega _h \rangle _{U_h} - ({\text {div}}u_h,q_h)&\gtrsim (\Vert u_h, \widehat{u}_h, \omega _h\Vert _{U_h} + \Vert {\text {div}}u_h \Vert _0)^2. \end{aligned}$$

#### Proof

For each element $T \in \mathcal {T}$ and each facet $F\subset \partial T$, there are matrix fields $S^F_0, S^F_1$, supported on *T*, with the following properties: on *T*, both $S^F_0, S^F_1$ are constant matrices in ${\mathbb {D}}$, their boundary trace $(S^F_i)_{nt}|_F$, for $i \in \{0, 1\}$, are constant unit-length vector fields on *F* that form a basis for the tangent space $n_F^\perp$, and $(S^F_i)_{nt}|_{F'}$ vanishes on all other facets $F' \ne F$ in $\mathcal {F}_h$. Such matrix fields are exhibited in [21, Lemma 5.1]. Given any $(u_h, \widehat{u}_h, \omega _h) \in U_h$, set

$$\begin{aligned} \tau _h^0&:= \sum _{T\in \mathcal {T}}\sum _{F\subset \partial T} \sum _{i \in \{0,1\}}- ( S^F_i : \varPi ^0{\text {dev}}(\nabla u_h - \kappa (\omega _h))) \; \lambda ^F S^F_i, \\ \tau _h^1&:= \sum _{T\in \mathcal {T}}\sum _{F\subset \partial T} \sum _{i \in \{0,1\}} \frac{1}{\sqrt{h}}\varPi ^0(\widehat{u}_h - u_h)_t\; S^F_i, \end{aligned}$$

where $\lambda _F$ is the linear barycentric coordinate function associated to the vertex opposite to the facet *F*. Since $\lambda ^FS^F_i$ has a vanishing *nt*-trace and $\varPi ^0{\text {dev}}(\nabla u_h - \kappa (\omega _h)) \in {\mathbb {D}}$, we see that $\tau _h = \gamma _0 \tau _h^0 + \gamma _1 \tau _h^1$, for any $\gamma _0,\gamma _1 \in {\mathbb {R}}$, is an element of $\varSigma _h$. Also set $q_h = -{\text {div}}u_h$, so that $-({\text {div}}u_h, q_h) = \Vert {\text {div}}u_h \Vert _0^2$. For these choices, (55) obviously holds as long as $\gamma _i$ is chosen independent of *h* and $\nu$. Indeed, such $\gamma _i$ can be chosen to also ensure that

$$\begin{aligned} \langle {\text {div}}\tau _h;\, u_h,\widehat{u}_h, \omega _h \rangle _{U_h} \gtrsim \Vert u_h,\widehat{u}_h, \omega _h\Vert _{U_h}^2, \end{aligned}$$

so that (56) also holds. This follows from an argument which (we omit and) is similar to that detailed in [20, Lemma 6.5], proceeding simply by appropriately combining Young and Cauchy–Schwarz inequalities. $\square$

The combined bilinear form of the MCS method (51) is given by

$$\begin{aligned} B(\sigma _h, u_h, \widehat{u}_h, \omega _h, p_h; \tau _h, v_h, \widehat{v}_h, \eta _h, q_h) :=&\nu ^{-1}(\sigma _h, \tau _h) + \langle {\text {div}}\tau _h; \, u_h,\widehat{u}_h, \omega _h \rangle _{U_h}\\&- \langle {\text {div}}\sigma _h;\, v_h,\widehat{v}_h, \eta _h \rangle _{U_h} \\&- ({\text {div}}u_h, q_h) -(p_h, {\text {div}}v_h) + c(\omega _h, \eta _h). \end{aligned}$$

Define a norm on the product space $S_h = \varSigma _h \times V_h \times \widehat{V}_h \times W_h \times Q_h$ by

$$\begin{aligned} \Vert \sigma _h, u_h, \widehat{u}_h, \omega _h, p_h\Vert _{S_h} := \nu ^{-1/2} ( \Vert \sigma _h\Vert _0 + \Vert p_h\Vert _0 ) + \nu ^{1/2} \Vert u_h, \widehat{u}_h, \omega _h\Vert _{\varepsilon }. \end{aligned}$$

#### Lemma 11

(Inf-sup condition for MCS method) For any $r = (\sigma _h, u_h, \widehat{u}_h, \omega _h, p_h) \in S_h$, be arbitrary, there exists an $s\in S_h$ such that

57 $$\begin{aligned} B(r; s)&\gtrsim \Vert r\Vert ^2_{S_h}, \quad \text { and} \end{aligned}$$

58 $$\begin{aligned} \Vert s \Vert _{S_h}&\lesssim \Vert r \Vert _{S_h}. \end{aligned}$$

#### Proof

We will find the required *s* as a sum of three terms, each in $S_h$, and each depending on the given *r*. The first term is set using $s^* = (\sigma _h, u_h, \widehat{u}_h, \omega _h, -p_h)$, for which we obviously have

59a $$\begin{aligned} B(r, s^*)&= \nu ^{-1} \Vert \sigma _h \Vert _0^2 + \nu h^2 \Vert {\text {div}}\omega _h\Vert _0^2,\end{aligned}$$

59b $$\begin{aligned} \Vert s^* \Vert _{S_h}&\lesssim \Vert r \Vert _{S_h}. \end{aligned}$$

The second term is ${\tilde{s}} = (\nu \tau _h, 0, 0, 0, \nu q_h) \in S_h$, where $\tau _h \in \varSigma _h$ and $q_h\in Q_h$ are as in Lemma 10 obtained using the given components $u_h, \widehat{u}_h, \omega _h$ of *r*. The lemma gives some ${\tilde{C}} > 0$ such that

60a $$\begin{aligned} B(r; {\tilde{s}})&= \nu ^{-1} (\sigma _h, \nu \tau _h) + \nu \langle {\text {div}}\tau _h; \, u_h,\widehat{u}_h, \omega _h \rangle _{U_h} - \nu ({\text {div}}u_h, q_h) \nonumber \\&\gtrsim (\sigma _h, \tau _h) + \nu \Big ( \Vert u_h, \widehat{u}_h, \omega _h\Vert ^2_{U_h} + \Vert {\text {div}}u_h \Vert _0^2\Big ), \end{aligned}$$

60b $$\begin{aligned} \Vert {\tilde{s}} \Vert _{S_h}^2&= \nu ^{-1}( \Vert \nu \tau _h\Vert _0^2 + \Vert \nu q_h\Vert _0^2) \le {\tilde{C}} \nu \Big ( \Vert u_h, \widehat{u}_h, \omega _h\Vert ^2_{U_h} + \Vert {\text {div}}u_h \Vert ^2_0\Big ). \end{aligned}$$

The third term is $s^\varDelta = (0, -\nu ^{-1}v_h, -\nu ^{-1}\widehat{v}_h, -\nu ^{-1} \eta _h, 0) \in S_h$ where $(v_h, \widehat{v}_h, \eta _h) \in U_h$ is as in Lemma 8 obtained using the given component $p_h$ of *r*. The lemma implies that ${\text {div}}v_h = p_h$ and

61a $$\begin{aligned} B(r; s^\varDelta )&= \nu ^{-1} \Vert p_h \Vert _0^2 - \nu ^{-1} \langle {\text {div}}\sigma _h;\, v_h,\widehat{v}_h, \eta _h \rangle _{U_h} + \nu ^{-1} c(\omega _h, \eta _h), \end{aligned}$$

61b $$\begin{aligned} \Vert s^\varDelta \Vert _{S_h}^2&= \nu \Vert \nu ^{-1}v_h, \nu ^{-1}\widehat{v}_h, \nu ^{-1} \eta _h \Vert _\varepsilon ^2 \lesssim \nu ^{-1}\Vert p_h\Vert _0^2. \end{aligned}$$

Note that to obtain the last inequality, we have also used Lemma 4.

Now letting $\beta > 0$, a constant yet to be chosen, put $s = \beta s^* + {\tilde{s}} + s^\varDelta$. Then, combining (59a), (60a) and (61a),

62 $$\begin{aligned} \begin{aligned} B(r; s)&\gtrsim \frac{\beta }{\nu }\Vert \sigma _h\Vert ^2_0 + \beta \nu h^2\Vert {\text {div}}\omega _h \Vert ^2_0 + \nu \Vert u_h, \widehat{u}_h, \omega _h\Vert ^2_{U_h}\\&\quad + \nu \Vert {\text {div}}u_h \Vert ^2_0 + \frac{1}{\nu }\Vert p_h \Vert _0^2 - (\rho _1 + \rho _2 + \rho _3), \end{aligned} \end{aligned}$$

where $\rho _1 = (\sigma _h, \tau _h), \rho _2 = - \nu ^{-1}\langle {\text {div}}\sigma _h;\, v_h,\widehat{v}_h, \eta _h \rangle _{U_h}, \; \rho _3 = \nu ^{-1} c(\omega _h, \eta _h)$. By (60b) and Young’s inequality,

$$\begin{aligned} \rho _1&\le \frac{{\tilde{C}}}{2 \nu } \Vert \sigma _h\Vert _0^2 + \frac{\nu }{2} \Big ( \Vert u_h, \widehat{u}_h, \omega _h\Vert ^2_{U_h} + \Vert {\text {div}}u_h \Vert ^2_0\Big ). \end{aligned}$$

To bound $\rho _2$, note that by Lemma 9, $\rho _2 \lesssim \nu ^{-1} \Vert \sigma _h \Vert _0 \Vert v_h,\widehat{v}_h, \eta _h\Vert _\varepsilon$, so by (61b), there is a $C^\varDelta >0$ such that $\rho _2 \le \nu ^{-1/2} \Vert \sigma _h \Vert _0 \left( \frac{1}{2} C^\varDelta \nu ^{-1} \Vert p_h \Vert _0^2\right) ^{1/2}$. Thus

$$\begin{aligned} \rho _2&\le \frac{C^\varDelta }{2 \nu } \Vert \sigma _h\Vert _0^2 + \frac{1}{4\nu } \Vert p_h\Vert _0^2. \end{aligned}$$

To bound $\rho _3$, we recall from Lemma 4 that $h\Vert {\text {div}}\eta _h \Vert _0 \lesssim \Vert v_h,\widehat{v}_h, \eta _h \Vert _{\varepsilon }$. Hence by (61b), there is a $C'>0$ such that $\rho _3 \le (\nu ^{1/2}h \Vert {\text {div}}\omega _h \Vert _0) \left( \frac{1}{2} C' \nu ^{-1} \Vert p_h \Vert _0^2 \right) ^{1/2}$, so

$$\begin{aligned} \rho _3 \le \frac{C'\nu }{2} h^2 \Vert {\text {div}}\omega _h \Vert _0^2 + \frac{1}{4 \nu } \Vert p_h \Vert _0^2. \end{aligned}$$

Using these estimates for $\rho _i$ in (62),

$$\begin{aligned} B(r; s)&\gtrsim \frac{2\beta - ({\tilde{C}} + C^\varDelta )}{2\nu } \Vert \sigma _h\Vert ^2_0 + \frac{2\beta - C'}{2}\nu h^2\Vert {\text {div}}\omega _h \Vert ^2_0 \\&\quad + \frac{\nu }{2} \Vert u_h, \widehat{u}_h, \omega _h\Vert ^2_{U_h} + \frac{\nu }{2} \Vert {\text {div}}u_h \Vert ^2_0 + \frac{1}{2\nu } \Vert p_h \Vert _0^2. \end{aligned}$$

Since ${\tilde{C}}, C^\varDelta$ and $C'$ are mesh-independent constants, choosing $\beta > \max ({\tilde{C}} + C^\varDelta , C')/2$ and recalling the norm equivalence of Lemma 4, we prove (57). Of course, inequality (58) follows from (59b), (60b), and (61b). $\square$

### Pressure Robust Error Analysis of MCS Scheme

In addition to the spaces $U_{{\text {reg}}}$ and $Q_{{\text {reg}}}$, the *a priori* error analysis will now also use a stress space with improved regularity, $\varSigma _{{\text {reg}}}:= \varSigma ^{{\text {sym}}} \cap H^1(\mathcal {T}, {\mathbb {D}})$. Note that the integrals in the terms defining $B(\sigma , u, \widehat{u}, \omega , p; \cdot )$ are well-defined for $\sigma \in \varSigma _{{\text {reg}}}$, $(u, u_t, \omega ) \in U_{{\text {reg}}}$, and $p\in Q_{{\text {reg}}}$, so $B(\cdot , \cdot )$ can be extended to such non-discrete arguments.

**Table 1:** Errors and estimated order of convergence (eoc) for the HDG method

$|\mathcal {T}|$	$\Vert \varepsilon ( u- u_h)\Vert _0$	eoc	$\Vert u - u_h\Vert _0$	eoc	$\Vert \omega - \omega _h\Vert _0$	eoc	$\Vert p - p_h\Vert _0$	eoc
63	$2.2.\,10^{-3}$	( – )	$1.9.\,10^{-4}$	( – )	$3.2.\,10^{-3}$	( – )	$2.1.\,10^{-1}$	( – )
504	$1.7.\,10^{-3}$	( 0.4 )	$8.4.\,10^{-5}$	( 1.2 )	$2.3.\,10^{-3}$	( 0.5 )	$1.2.\,10^{-1}$	( 0.9 )
4032	$9.3.\,10^{-4}$	( 0.9 )	$2.4.\,10^{-5}$	( 1.8 )	$1.2.\,10^{-3}$	( 0.9 )	$6.1.\,10^{-2}$	( 0.9 )
32256	$5.38.\,10^{-4}$	( 0.8 )	$8.0.\,10^{-6}$	( 1.6 )	$6.6.\,10^{-4}$	( 0.9 )	$3.1.\,10^{-2}$	( 1.0 )
258048	$2.8.\,10^{-4}$	( 0.9 )	$2.3.\,10^{-6}$	( 1.8 )	$3.5.\,10^{-4}$	( 0.9 )	$1.6.\,10^{-2}$	( 1.0 )
2064384	$1.4.\,10^{-4}$	( 1.0 )	$6.3.\,10^{-7}$	( 1.9 )	$1.8.\,10^{-4}$	( 1.0 )	$7.8.\,10^{-3}$	( 1.0 )

#### Lemma 12

(Consistency of the MCS method) Assume that the exact solution $(\sigma , u,p)$ of (7) fulfills the regularity assumption $(u, u_t, \omega ) \in U_{{\text {reg}}}$ and $(\sigma , p) \in \varSigma _{{\text {reg}}}\times Q_{{\text {reg}}}$, where $\omega = {\text {curl}}(u)$. Let $(\sigma _h, u_h, \widehat{u}_h, \omega _h, p_h) \in S_h$ be the solution of (51) and let $(\tau _h, v_h, \widehat{v}_h, \eta _h, q_h) \in S_h$ be an arbitrary test function. Then

63 $$\begin{aligned} B(\sigma - \sigma _h, u-u_h, u_t - \widehat{u}_h, \omega - \omega _h, p - p_h; \tau _h, v_h, \widehat{v}_h, \eta _h, q_h) = 0. \end{aligned}$$

#### Proof

Since $\sigma$ is symmetric we have that $\sigma : \kappa (\eta _h)=0$. Next, using the regularity assumptions, starting from (53), we get

$$\begin{aligned} -\langle {\text {div}}\sigma ;\,&v_h, \widehat{v}_h, \eta _h\rangle _{U_h} - ({\text {div}}v_h, p_h) = (\sigma - pI :\nabla v_h)_\mathcal {T}- (\sigma _{nt} ,\cdot (v_h-\widehat{v}_h)_t)_{\partial \mathcal {T}}\\&= -({\text {div}}(\sigma - pI), v_h)_\mathcal {T}- (\sigma _{nt}, (v_h-\widehat{v}_h)_t)_{\partial \mathcal {T}} +((\sigma - pI)n, v_h )_{\partial \mathcal {T}}\\&= -({\text {div}}(\sigma - pI), v_h)_\mathcal {T}+ (\sigma _{nt}, \widehat{v}_h)_{\partial \mathcal {T}} - ((\sigma - pI)_{nn}, (v_h)_n)_{\partial \mathcal {T}} \\&= -({\text {div}}(\sigma - pI), v_h)_\mathcal {T}+ \sum _{F\in \mathcal {F}} (\llbracket {{\sigma }}\rrbracket _{nt}, \widehat{v}_h)_F - (\llbracket {{(\sigma - pI)}}\rrbracket _{nn}, (v_h)_n)_F \\&= -({\text {div}}(\sigma - pI), v_h)_\mathcal {T}+ \int _{\varGamma _N}(\sigma - pI)_{nn} (v_h)_n - \sigma _{nt} \widehat{v}_h \mathop {{\textrm{d}} s}\\&= -({\text {div}}(\sigma - pI), v_h) = (f, v_h), \end{aligned}$$

where the boundary integral vanished using (2f) given on $\varGamma _N$. Next, since $\nu ^{-1}\sigma =\varepsilon (u)=\nabla u - \kappa (\omega )$ we have

$$\begin{aligned} \nu ^{-1} (\sigma , \tau _h)&+ \langle {\text {div}}\tau _h; \,u, \widehat{u}, \omega \rangle _{U_h} \\&= \nu ^{-1} (\sigma , \tau _h) -(\tau _h, \nabla u -\kappa (\omega ))_\mathcal {T}+ (\tau _{nt}, (u - u_t)_t)_{\partial \mathcal {T}} = 0. \end{aligned}$$

The final remaining term in the bilinear form is also zero since $({\text {div}}u, q_h) = 0$ as the exact solution is divergence free. $\square$

#### Theorem 2

(Error estimate for the MCS method) Assume that the exact solution $(\sigma , u,p)$ of (7) fulfills the regularity assumption $(u, u_t, \omega ) \in U_{{\text {reg}}}$ and $(\sigma , p) \in \varSigma _{{\text {reg}}}\times Q_{{\text {reg}}}$, where $\omega = {\text {curl}}(u)$. Let $(u_h, \widehat{u}_h, \omega _h) \in U_h$ and $(\sigma _h,p_h) \in \varSigma _h \times Q_h$ be the solution of (51). Then we have the pressure robust error estimate

64 $$\begin{aligned} \nu ^{-1}\Vert \sigma - \sigma _h\Vert _0 + \Vert u-u_h, u_t - \widehat{u}_h, \omega - \omega _h\Vert _{\varepsilon } \lesssim h \Vert u\Vert _{H^2(\mathcal {T})} . \end{aligned}$$

Furthermore, the pressure error can be bounded by

65 $$\begin{aligned} \nu ^{-1}\Vert p - p_h\Vert _0 \lesssim h \big (\Vert u\Vert _{H^2(\mathcal {T})} + \nu ^{-1}\Vert p\Vert _{H^1(\mathcal {T})}\big ). \end{aligned}$$

**Table 2:** Errors and estimated order of convergence (eoc) for the MCS method

$|\mathcal {T}|$	$\Vert \varepsilon (u - u_h)\Vert _0$	eoc	$\Vert u - u_h\Vert _0$	eoc	$\Vert \sigma - \sigma _h\Vert _0$	eoc	$\Vert \omega - \omega _h\Vert _0$	eoc	$\Vert p - p_h\Vert _0$	eoc
63	$2.6.\,10^{-3}$	( – )	$2.1.\,10^{-4}$	( – )	$4.0.\,10^{-7}$	( – )	$3.2.\,10^{-3}$	( – )	$2.1.\,10^{-1}$	( – )
504	$1.9\,10^{-3}$	( 0.4 )	$1.0.\,10^{-4}$	( 1.0 )	$2.9.\,10^{-7}$	( 0.5 )	$2.2.\,10^{-3}$	( 0.5 )	$1.2.\,10^{-1}$	( 0.9 )
4032	$1.0\,10^{-4}$	( 0.9 )	$2.5.\,10^{-5}$	( 2.0 )	$1.5.\,10^{-7}$	( 1.0 )	$1.1.\,10^{-3}$	( 1.0 )	$6.1.\,10^{-2}$	( 0.9 )
32256	$6.0\,10^{-4}$	( 0.7 )	$7.8.\,10^{-6}$	( 1.7 )	$7.9.\,10^{-8}$	( 0.9 )	$6.1.\,10^{-4}$	( 0.9 )	$3.1.\,10^{-2}$	( 1.0 )
258048	$3.1\,10^{-4}$	( 1.0 )	$2.0.\,10^{-6}$	( 1.9 )	$4.0.\,10^{-8}$	( 1.0 )	$3.1.\,10^{-4}$	( 1.0 )	$1.6.\,10^{-2}$	( 1.0 )
2064384	$1.5\,10^{-4}$	( 1.0 )	$5.2.\,10^{-7}$	( 2.0 )	$2.0.\,10^{-8}$	( 1.0 )	$1.5.\,10^{-4}$	( 1.0 )	$7.8.\,10^{-3}$	( 1.0 )

#### Proof

As in the proof of Theorem 1, let $E = (\sigma -\sigma _h, u-u_h, u_t - \widehat{u}_h, \omega -\omega _h, p - p_h),$ $E_h = (I_\varSigma \sigma -\sigma _h, I_Vu-u_h, I_{\hat{V}}u_t - \widehat{u}_h, I_W\omega -\omega _h, I_Qp-p_h)$, and let the interpolation error be ${\mathcal {E}} = E - E_h$. Now, using Lemma 11, choose $s = (\tau _h, v_h, \widehat{v}_h, \eta _h, q_h)$ such that

$$\begin{aligned} \Vert E_h\Vert _{S_h}&\lesssim \frac{B( E_h;s)}{\Vert s\Vert _{S_h}}. \end{aligned}$$

By the consistency of the MCS formulation (63) we have

$$\begin{aligned} B(E_h;s) = B(E - \mathcal {E};s) = B(\mathcal {E};s). \end{aligned}$$

Hence, if we prove that

66 $$\begin{aligned} B(\mathcal {E}; s) \lesssim \nu ^{1/2}h \Vert u\Vert _{H^2(\mathcal {T})} \Vert s\Vert _{S_h}, \end{aligned}$$

then $\Vert E_h \Vert _{S_h} \lesssim \nu ^{1/2}h \Vert u\Vert _{H^2(\mathcal {T})}$, which is enough to yield the stated pressure-independent estimate (64): indeed, letting ${\bar{E}}:= (\sigma -\sigma _h, u-u_h, u_t - \widehat{u}_h, \omega -\omega _h, 0)$, ${\bar{E}}_h:= (I_\varSigma \sigma -\sigma _h, I_Vu-u_h, I_{\hat{V}}u_t - \widehat{u}_h, I_W\omega -\omega _h, 0)$, and $\bar{{\mathcal {E}}} = {\bar{E}} - {\bar{E}}_h$, we would then have

67 $$\begin{aligned} \Vert {\bar{E}} \Vert _{S_h} \le \Vert \bar{{\mathcal {E}}} \Vert _{S_h} + \Vert {\bar{E}}_h \Vert _{S_h} \le \Vert \bar{{\mathcal {E}}} \Vert _{S_h} + \Vert E_h \Vert _{S_h} \le \nu ^{1/2} h \Vert u\Vert _{H^2(\mathcal {T})}, \end{aligned}$$

using the interpolation estimates (28)–(29) to bound $\Vert \bar{{\mathcal {E}}} \Vert _{S_h}$. Inequality (67) obviously implies (64). Therefore we focus on proving (66) and proceed to separately inspect each term forming its left hand side.

Let $\mathcal {E}^j$ with $j \in \{\sigma , u, \widehat{u}, \omega , p\}$ denote the corresponding components of the interpolation error. Then (53) implies

$$\begin{aligned} \langle {\text {div}}\tau _h; \mathcal {E}^u, \mathcal {E}^{\widehat{u}}, \mathcal {E}^{\omega }\rangle&= (\tau _h, \kappa (\mathcal {E}^{\omega }) - \nabla \mathcal {E}^u)_\mathcal {T}+ ((\tau _h)_{nt}, (\mathcal {E}^u - \mathcal {E}^{\widehat{u}})_t)_{\partial \mathcal {T}}. \end{aligned}$$

As $(\tau _h)_{nt}$ is constant on each facet, we can insert $\varPi ^0$ in the last term, so several applications of the Cauchy–Schwarz inequality with $h^{1/2}$ and $h^{-1/2}$ weights for the boundary terms yields

68 $$\begin{aligned} \langle {\text {div}}\tau _h;\, \mathcal {E}^u, \mathcal {E}^{\widehat{u}}, \mathcal {E}^{\omega }\rangle&\lesssim \big ( \Vert \tau _h\Vert _0 + h^{1/2}\Vert (\tau _h)_{nt}\Vert _{\partial \mathcal {T}}\big )(\Vert \mathcal {E}^u,\mathcal {E}^{\widehat{u}}\Vert _{\nabla } + \Vert \kappa (\mathcal {E}^{\omega })\Vert _0)\nonumber \\&\lesssim \Vert \tau _h\Vert _0 h \Vert u\Vert _{H^2(\mathcal {T})}, \end{aligned}$$

where we used (54) again and the interpolation estimate (28) in the last step.

Next consider the symmetrically opposite term in *B*. Since $\nabla v_h\in P^0(\mathcal {T})$ and $\mathcal {E}^{\sigma }$ is orthogonal to facet-wise and element-wise constant functions [see (26)–(27)], we have

$$\begin{aligned} -\langle {\text {div}}\mathcal {E}^{\sigma }; v_h, \widehat{v}_h, \eta _h\rangle _{U_h}&= (\mathcal {E}^\sigma , \nabla v_h - \kappa (\eta _h))_\mathcal {T}- (\mathcal {E}^\sigma _{nt}, (v_h - \widehat{v}_h)_t)_{\partial \mathcal {T}} \\&= -(\mathcal {E}^\sigma , (I-\varPi ^0)\kappa (\eta _h))_\mathcal {T}- (\mathcal {E}^\sigma _{nt}, (I-\varPi ^0)(v_h - \widehat{v}_h)_t)_{\partial \mathcal {T}} \\&\lesssim \Vert \mathcal {E}^\sigma \Vert _0 ~\Vert v_h, \widehat{v}_h, \eta _h\Vert _{\varepsilon } + h^{1/2}\Vert \mathcal {E}^\sigma _{nt}\Vert _{\partial \mathcal {T}} \Vert v_h,\widehat{v}_h \Vert _\nabla . \end{aligned}$$

where on the right hand side of the last inequality, the first term is obtained using (20b) and Lemma 4, while the second term is obtained using (48). Thus, the interpolation estimate (29) and Lemma 2 imply

69 $$\begin{aligned} \langle {\text {div}}\mathcal {E}^{\sigma }; v_h, \widehat{v}_h, \eta _h\rangle _{U_h} \lesssim \nu h \Vert u\Vert _{H^2(\mathcal {T})} \Vert v_h, \widehat{v}_h, \eta _h\Vert _{\varepsilon }. \end{aligned}$$

The remaining terms are easy: by Cauchy–Schwarz inequality,

70 $$\begin{aligned} \nu ^{-1}(\mathcal {E}^\sigma , \tau _h) \lesssim h \Vert u\Vert _{H^2(\mathcal {T})} \Vert \tau _h \Vert _0, \end{aligned}$$

and by the definition of $I_W, I_Q$ and (25),

71 $$\begin{aligned} ({\text {div}}\mathcal {E}^{\omega }, {\text {div}}\eta _h )&= 0,\quad ({\text {div}}\mathcal {E}^u, q_h) = 0, \quad \text {and} \quad (\mathcal {E}^p, {\text {div}}v_h) = 0, \end{aligned}$$

where the last equation is due to ${\text {div}}v_h\in P^0(\mathcal {T})$. Summing up (68), (69), (70), and (71), we prove (66), and hence (64).

The pressure error estimate (65) follows along the same lines as in the proof of Theorem 1. $\square$

## Numerical Examples

In this last section we present a simple numerical example to provide a practical illustration of the theoretical asymptotic convergence rates as well as to compare the two new methods we presented. Both methods were implemented within the finite element library NGSolve/Netgen (see [32, 33] and www.ngsolve.org). Testfiles and our computational results are available at [28].

**Fig. 3:**
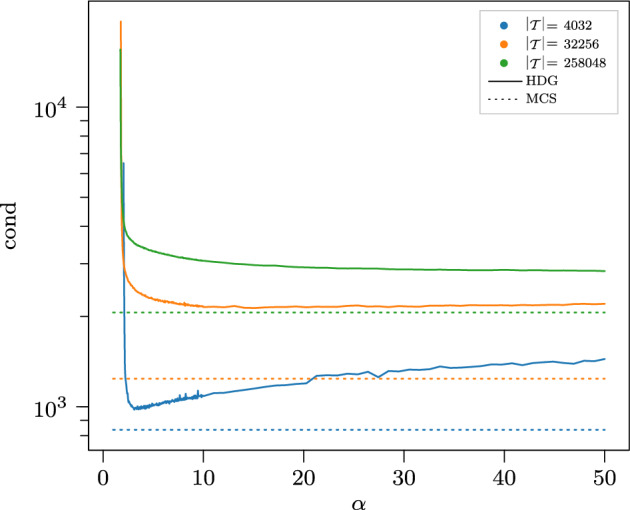
Approximate condition numbers of the corresponding *A* blocks of the HDG (solid lines) and the MCS (dotted lines in the same color) method on different meshes. Different values of $\alpha$ on the x axis and approximate condition number (${\text {cond}}$) on the y axis

The computational domain is given by $\varOmega = (0,1)^3$ and the velocity field is driven by the volume force determined by $f = -{\text {div}}\sigma + \nabla p$ with the exact solution given by

$$\begin{aligned} \sigma&= \nu \varepsilon ({\text {curl}}(\psi ,\psi ,\psi )), \quad \text {and} \quad p := x^5 + y^5 +z^5 - \frac{1}{2}. \end{aligned}$$

Here $\psi :=x^2(x-1)^2y^2(y-1)^2z^2(z-1)^2$ defines a given potential and we choose the viscosity $\nu = 10^{-4}$. While this would lend itself to homogenous Dirichlet conditions being prescribed on the whole boundary, as we assume $|\varGamma _N|>0$ throughout the paper, we instead opt to impose non-homogenous Neumann conditions on $\varGamma _N:=\{0\}\times (0,1)\times (0,1)$ and homogenous Dirichlet conditions only on $\varGamma _D:=\partial \varOmega \setminus \varGamma _N$. Note that this requires the additional source terms $\int _{\varGamma _N}(\sigma _{nn} - p) (v_h)_n\mathop {{\textrm{d}} s}$ and $\int _{\varGamma _N}\sigma _{nt}\widehat{v}_h\mathop {{\textrm{d}} s}$ to be provided as data for the methods.

***Convergence*** An initial, relatively coarse mesh was generated and then refined multiple times. With the larger problem size on finer meshes in mind, we used a GMRes Krylov space solver preconditioned by an auxiliary space method using a lowest order conforming $H^1$ space (see e.g., [16], and for details specific to the MCS case, see [22]) with relative tolerance of $10^{-14}$. Errors measured in different norms and their estimated order of convergence (eoc) are listed in Table 1 for the HDG method and Table 2 for the MCS method. For the HDG method we chose the stabilization parameter $\alpha =6$. As predicted by the analysis from Theorem 1 and Lemma 2, the velocity error measured in the seminorm $\Vert \varepsilon (u - u_h)\Vert _0$, the $L^2$-norm of the vorticity, and the pressure errors converge at optimal linear order. Furthermore, for the MCS method, we also observe optimal convergence for the stress error. In addition, we also plotted the $L^2$-norm error of the velocity. From an Aubin-Nitsche argument one may expect a higher order of convergence whenever the dual problem shows enough regularity [3, 21]. Not surprisingly therefore, we observe quadratic convergence for the $L^2$-norm of the velocity error for both methods.

***Condition numbers*** For both HDG and MCS method, after static condensation within the $(u_h,\widehat{u}_h, \omega _h)$- or $(\sigma _h, u_h, \widehat{u}_h, \omega _h)$-block of the finite element matrix respectively, we obtain a symmetric and positive definite diagonal block, which we simply refer to here as the “*A*”-blocks of the respective methods. (Of course, due to the incompressibility constraint, the entire system is still of saddle point structure.) Both the *A* blocks have the same non-zero structure and are expected to have condition number $\mathcal {O}(h^{-2})$, but they discretize slightly different operators, namely $\varepsilon$ for the HDG method, and ${\text {dev}}(\varepsilon )$ for the MCS method. As $\varepsilon (u) = {\text {dev}}(\varepsilon (u)) + \frac{1}{3}{\text {div}}(u) {\text {I}}$ and the true solution is divergence-free, adding the (consistent) term $\frac{1}{3}{\text {div}}u_h {\text {div}}v_h$ to the MCS bilinear form yields an *A* block that is directly comparable to the one of the HDG method. In Fig. 3 we show approximate condition numbers (${\text {cond}}$) of said *A* blocks for some of the meshes used in the previous computations and different values of $\alpha$ in $a^{{\text {hdg}}}$. We see that in addition to the MCS method not being dependent on any stabilization parameter in the first place, there appears to be no possible choice of $\alpha$ that would make the HDG method’s *A* block better conditioned than that of the MCS method.

## Data Availability

The datasets and scripts to generate the data with the finite element library NGSolve www.ngsolve.org are available from the corresponding author and at [28].
